# The Behavioral Biology of Teams: Multidisciplinary Contributions to Social Dynamics in Isolated, Confined, and Extreme Environments

**DOI:** 10.3389/fpsyg.2019.02571

**Published:** 2019-11-21

**Authors:** Lauren Blackwell Landon, Grace L. Douglas, Meghan E. Downs, Maya R. Greene, Alexandra M. Whitmire, Sara R. Zwart, Peter G. Roma

**Affiliations:** ^1^Behavioral Health & Performance Laboratory, Biomedical Research and Environmental Sciences Division, Human Health and Performance Directorate, KBR/NASA Johnson Space Center, Houston, TX, United States; ^2^Advanced Food Technology, Human Systems Engineering and Development Division, Human Health and Performance Directorate, NASA Johnson Space Center, Houston, TX, United States; ^3^Human Physiology, Performance, Protection, and Operations Laboratory, Biomedical Research and Environmental Sciences Division, Human Health and Performance Directorate, KBR/NASA Johnson Space Center, Houston, TX, United States; ^4^Usability Testing and Analysis Facility, Human Systems Engineering and Development Division, Human Health and Performance Directorate, KBR/NASA Johnson Space Center, Houston, TX, United States; ^5^Human Factors and Behavioral Performance Element, Human Research Program, NASA Johnson Space Center, Houston, TX, United States; ^6^Nutritional Biochemistry Laboratory, Biomedical Research and Environmental Sciences Division, Human Health and Performance Directorate, University of Texas Medical Branch/NASA Johnson Space Center, Houston, TX, United States

**Keywords:** social dynamics, extreme environment, neurobiology, behavioral health, team performance, multidisciplinary

## Abstract

Teams in isolated, confined, and extreme (ICE) environments face many risks to behavioral health, social dynamics, and team performance. Complex long-duration ICE operational settings such as spaceflight and military deployments are largely closed systems with tightly coupled components, often operating as autonomous microsocieties within isolated ecosystems. As such, all components of the system are presumed to interact and can positively or negatively influence team dynamics through direct or indirect pathways. However, modern team science frameworks rarely consider inputs to the team system from outside the social and behavioral sciences and rarely incorporate biological factors despite the brain and associated neurobiological systems as the nexus of input from the environment and necessary substrate for emergent team dynamics and performance. Here, we provide a high-level overview of several key neurobiological systems relevant to social dynamics. We then describe several key components of ICE systems that can interact with and on neurobiological systems as individual-level inputs influencing social dynamics over the team life cycle—specifically food and nutrition, exercise and physical activity, sleep/wake/work rhythms, and habitat design and layout. Finally, we identify opportunities and strategic considerations for multidisciplinary research and development. Our overarching goal is to encourage multidisciplinary expansion of team science through (1) prospective horizontal integration of variables outside the current bounds of team science as significant inputs to closed ICE team systems and (2) bidirectional vertical integration of biology as the necessary inputs and mediators of individual and team behavioral health and performance. Prospective efforts to account for the behavioral biology of teams in ICE settings through an integrated organizational neuroscience approach will enable the field of team science to better understand and support teams who work, live, serve, and explore in extreme environments.

Teams that work, live, and serve in isolated, confined, and extreme (ICE) environments face many threats to behavioral health, social dynamics, and team performance over time (Landon et al., 2018). In the prototypical long-duration ICE environment—space exploration—as well as military deployments, remote work outposts, and other high-risk operational settings, teams must adapt to multiple interacting risks from the surrounding external environment, the constructed operational environment, the social environment, and individual-level vulnerabilities (Goswami et al., 2012; Roma and Bedwell, 2017).

In recognition of the critical and increasing importance of team-based work throughout society, including ICE operations, the field of team science has experienced rapid growth in recent years (DeChurch et al., 2018; Goodwin et al., 2018). Led largely by the Industrial/Organizational (I/O) subfield of Psychology, an appreciation for the complexity of teams in operational environments has enabled the innovative integration of theories, models, methods, and metrics from engineering and computer science, sociology, and other fields within the social sciences to enrich the understanding of social behavior and team performance. One of the major conceptual innovations that has come to define the field of team science is the Input-Mediator-Outcome-Input model of team dynamics (IMOI; Ilgen et al., 2005). Inspired by general systems theory, the IMOI model is a framework of how teams operate and change over time. The model is conceptualized as a flow from inputs (I) to mediators (M) to outputs (O), which then become inputs (I) for subsequent team performance cycles. Individual-level inputs include factors such as the team members’ respective personalities, knowledge, skills, abilities, and learning histories. Team-level inputs include group size, composition, roles, and leadership structure. Organizational-level inputs include the industry (e.g., corporate, military, and athletic) and operational context (e.g., office, virtual, and field site). Together, these inputs contribute to and interact with multiple emergent mediating phenomena that influence social dynamics, team performance outputs, and organizational outcomes. Mediators include team affective states (e.g., cohesion, confidence, and trust), behavioral processes (e.g., transition, action, and interpersonal behaviors), and cognitive processes (e.g., team learning, shared mental models, and transactive memory systems; Kozlowski and Ilgen, 2006; Fiore et al., 2015). Outputs include individual- and team-level performance, health and well-being, and organizational outcomes such as mission success, safety, and profitability. As a mission continues over time, the team repeats these performance episodes, with the outputs of each episode feeding back to shape the team’s mediating processes and states while becoming a contextual input for the next episode.

Although the structure of the IMOI model is largely agnostic to content, its manifestation within team science quite naturally focuses on input and mediator variables from the social and behavioral sciences from which it originated. However, at its most extreme, ICE operational settings are fully closed systems with tightly coupled, or highly interconnected, components (Perrow, 1984), i.e., fully autonomous microsocieties within isolated ecosystems involving far more than just the psychological processes of the inhabitants (Brady, 1990, 2005; Gitelson et al., 2003; Anker, 2005; Emurian et al., 2009; Checinska et al., 2015). Tightly coupled systems are those in which an unexpected occurrence can have an immediate and pervasive impact on the other parts of the system (Perrow, 1984). Systems with redundancy and flexibility between components, including input from outside the system, allow the system to be more resilient to disruptions; however, complexity of the system can also increase risk. A fully closed system with no outside input has even less flexibility than tightly coupled systems and potentially greater ripple effects of a disruption throughout the system. Insofar as ICE mission environments are closed systems, they are inherently “multidisciplinary” in that all components of the system—regardless of their scientific origins—can interact and potentially influence team dynamics through direct or indirect pathways. Thus, a primary goal of this article is to highlight several critical components of ICE mission environments that are outside the traditional bounds of team research, and how they may impact social dynamics and team performance over time as individual inputs in the IMOI model. Specifically, we discuss food and nutrition, exercise and physical activity, sleep/wake/work rhythms, and habitat design and layout. The purpose of this review is to encourage multidisciplinary horizontal integration of team science with fields relevant to ICE environments whose primary focus is not behavior, cognition, and social dynamics, but whose topics of focus can indirectly and directly impact team performance as individual-level inputs.

Our discussion of multidisciplinary contributions to social dynamics in ICE environments is firmly rooted in biology, on the premise that the brain is the nexus of individual-level inputs in the IMOI or any model of human functioning and thus worthy of systematic consideration in the science of teams. However, this emphasis on biological mechanisms is explicitly on inclusion and integration, not radical reductionism attempting to define behavioral, cognitive, and social phenomena as exclusively neurobiological (Ashkanasy et al., 2014). That said, even if the brain and associated neurobehavioral systems are not sufficient to define team phenomena, they are the necessary substrate from which team processes and social dynamics emerge (Krakauer et al., 2017; Killeen, 2018). Despite this, the proximal biological mechanisms of team performance and adaptation to extreme environments have received relatively little attention within team science (Golden et al., 2018; Maynard et al., 2018; Salas et al., 2018). This may be an artifact of I/O Psychology’s extension to “higher” levels of analysis, building off Psychology’s focus on behavior and cognition in individuals and small groups to incorporate multi-level frameworks including multi-team systems, organizations, cultures, societies, and related constructs (Kozlowski and Klein, 2000; Ilgen et al., 2005). By contrast, the subfield of Biological Psychology (including Social Neuroscience) shares I/O’s core interest in behavior and cognition in individuals and small groups but extends into “lower” levels of analysis, drawing from the natural sciences in the biomedical tradition to incorporate factors such as physiological systems, brain circuits, neurochemicals, and genetics. Consequently, another goal of this article is to not only encourage expanding team science through horizontal integration across disciplines but also encourage bidirectional vertical integration of multiple levels of analysis from the molecular through the societal in support of further development of an “organizational neuroscience” (cf. Becker and Cropanzano, 2010; Lee et al., 2012; Foxall, 2014a,b; Murray and Antonakis, 2019; Figure 1). Such an integrated approach is especially relevant for the application of team science to the tightly coupled closed systems of long-duration ICE settings, where the behavioral biology of teams is effectively defined by both horizontal and vertical factors continuously interacting and converging on the brain to influence individual and team behavioral health and performance over time (Figure 2).

**Figure 1:**
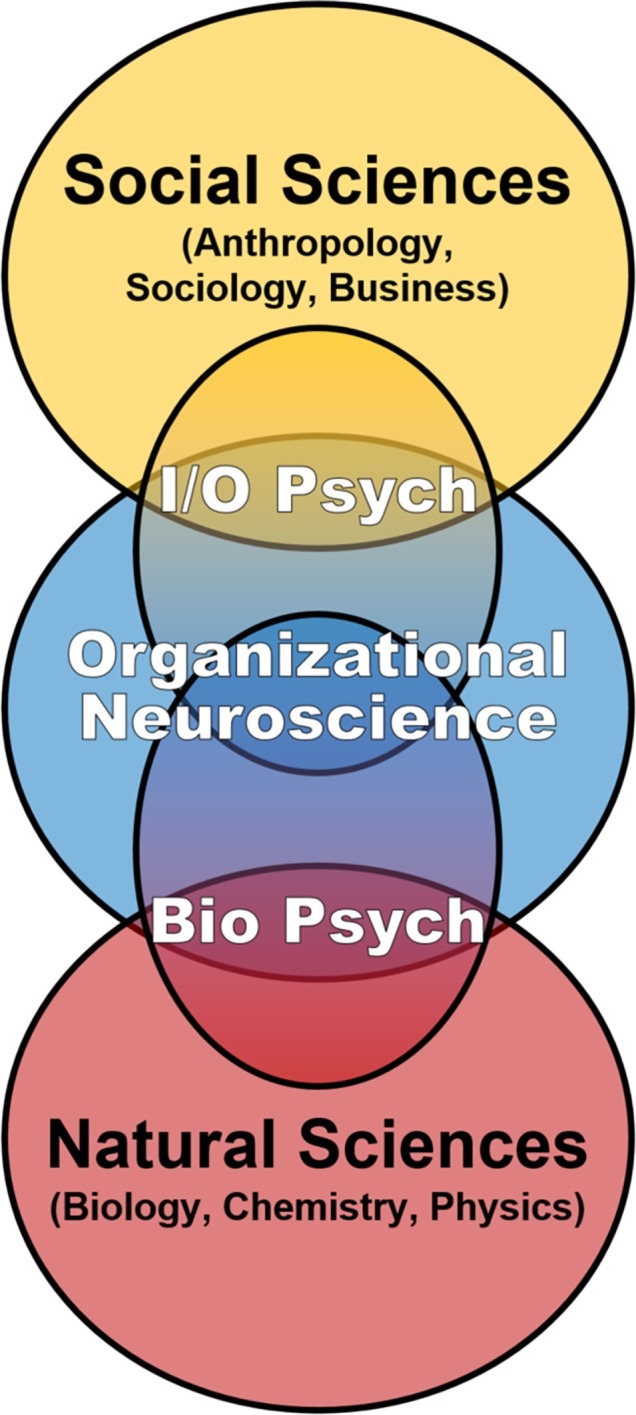
Team science is currently dominated by the Industrial/Organizational subfield of Psychology, which skillfully integrates multi-level frameworks, concepts, and methods from the social sciences. We support bidirectional vertical expansion of team science toward further development of organizational neuroscience that includes multiple biological levels of analysis to more fully understand individual and team functioning over time in the inherently integrated setting of isolated, confined, and extreme environments.

**Figure 2:**
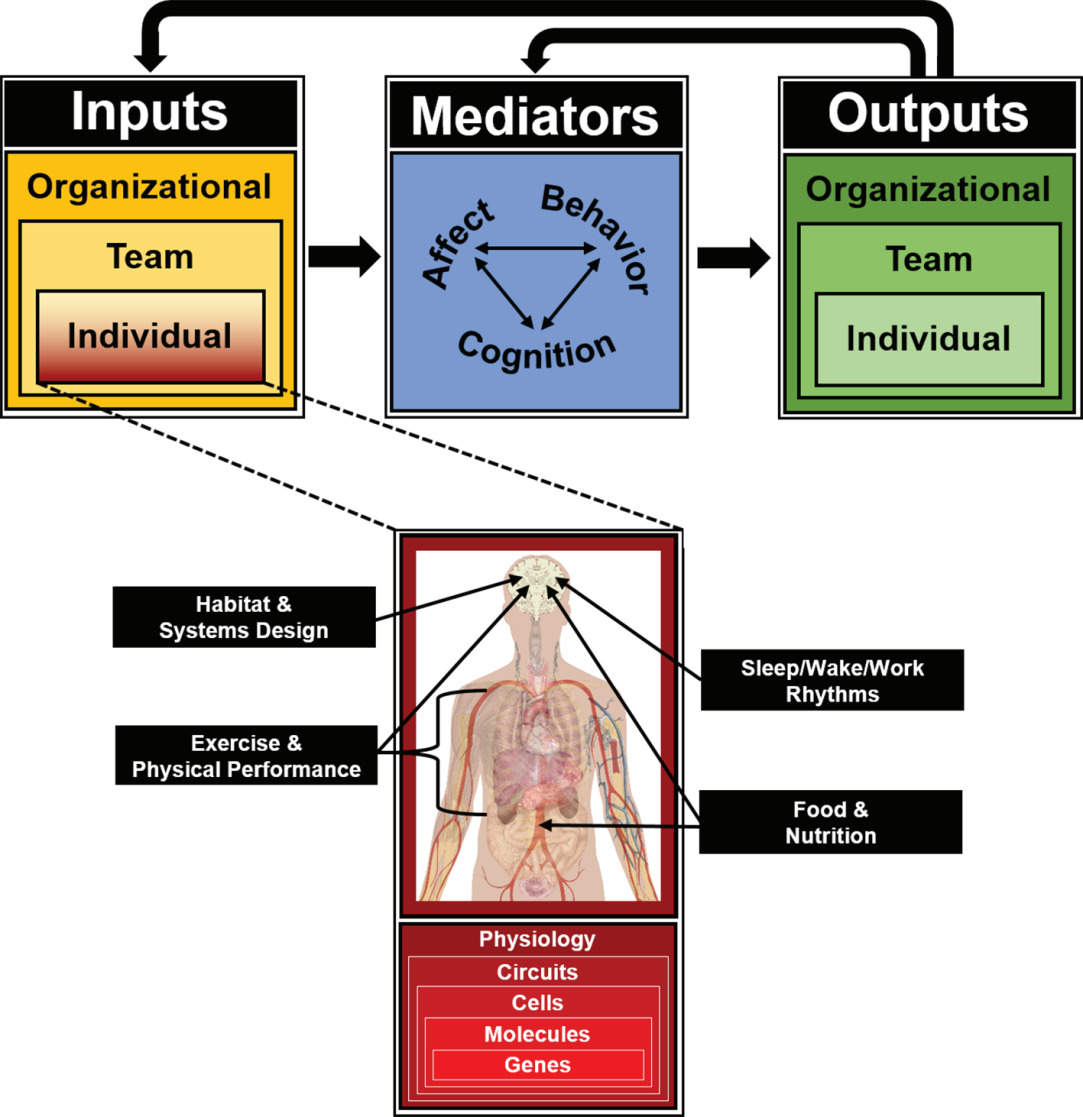
Input-Mediator-Output-Input (IMOI) model of team function for isolated, confined, and extreme operational environments, adapted to reflect “horizontal” integration of individual input variables inherent to ICE settings but outside the founding disciplines of team science, and “vertical” integration of multiple biological levels of analysis as targets and substrates for all individual inputs to the team system as an integrated approach toward the behavioral biology of teams.

The following sections first provide a selective overview of several core neurobiological systems relevant to individual and team behavioral health and performance within the closed systems of isolated, confined, and extreme operational environments. We then describe several key components of ICE systems that can interact with and on individual neurobiological systems to affect social dynamics—specifically food and nutrition, exercise and physical activity, sleep/wake/work rhythms, and habitat design and layout. Using long-duration space exploration missions as a prototypical ICE team setting, we consider how each of these disciplines may inform team researchers to understand ICE teams from a systemic, biological perspective, particularly as social dynamics develop over the life cycle of a team. Finally, we discuss opportunities and strategic considerations for prospective integrated multidisciplinary team research for ICE environments.

## Core Neurobehavioral Mechanisms for Isolated, Confined, and Extreme Teams

Humans are demonstrably capable of thriving in a wide variety of environments, so it comes as no surprise that we have evolved complex neurobehavioral systems for perceiving, responding, and adapting to the physical and social contexts in which we live, work, and explore. However, by their very nature, ICE environments are only extreme because they diverge in many ways from environments in which humans naturally thrive, and indeed, the brain provides an extraordinarily rich target for all components of ICE environments to profoundly affect individual and team behavioral health, performance, and social dynamics. To this end, we provide a brief and simplified overview of selected neurobiological systems underlying individual and team adaptation to ICE environments. These systems serve not only as both direct and indirect targets of the various input variables described in subsequent sections of this article but also as potential targets for countermeasure development to monitor, maintain, and enhance team dynamics in ICE mission settings.

To help guide the discussion, we refer to the National Institute of Mental Health’s (NIMH) Research Domain Criteria (RDoC) framework (Cuthbert and Kozak, 2013)^1^. Although the primary goal of RDoC is to elucidate the nature of mental health and illness, it does so not through a traditional symptom/category-based clinical diagnostic approach but rather by defining the degree of (dys) function of core overlapping neurobehavioral systems (“domains”) applicable to all individuals, teams, and situations (Clark et al., 2017). The six domains include the physical functions of the *Arousal and Regulatory Systems* (including sleep-wakefulness and circadian rhythms) and *Sensorimotor* (including action initiation and inhibition) domains, as well as the psychological and social domains of *Negative Valence* (including fear, anxiety, and loss), *Positive Valence* (including reward responsiveness and reinforcement), *Cognitive* (including memory and cognitive control), and *Social Processes* (including affiliation and communication). Although RDoC is an evolving framework continuously undergoing review and revision as the underlying science advances, a defining feature is that each domain’s function is characterized through multiple levels of influence from genes, molecules, cells, circuits, and physiological systems through to observable behaviors. For the sake of brevity, we focus our discussion on behavioral and physiological outputs, primary brain circuits and structures, and associated neurochemicals underlying key constructs within and across domains, and how they may relate to IMOI team systems in extreme environments. Subsequent sections describing team implications of food and nutrition, exercise and activity, sleep/wake/work rhythms, and habitat design include relevant biological mechanisms, and we consider potential pathways by which those factors may impact core neurobehavioral systems as individual-level inputs affecting team behavioral health and performance in ICE environments.

### Arousal/Regulatory and Sensorimotor Systems

The systems of the arousal/regulatory and sensorimotor domains serve essential biobehavioral functions, most notably sleep-wakefulness rhythms and physical movement. In a team mission context, wakefulness and sufficient attentional and physical capacity are required for functional presence and participation in any team processes and activities. Beyond presence vs. absence, individual differences in sleep-wake rhythms and interactions with mission schedules and features of the constructed environment can impact team dynamics as individual- or team-level inputs to the IMOI model. Biologically, perhaps the most critical brain structure regulating sleep/wake rhythms is the suprachiasmatic nucleus (SCN) within the hypothalamus. Light-sensitive cells in the retina project the excitatory neurotransmitter glutamate directly to the SCN, which help entrain the SCN’s rhythm as the brain’s “master clock” governing release of melatonin from the pineal gland to promote sleep (Ebling, 1996; Altun and Ugur-Altun, 2007; Dubocovich, 2007). The SCN also receives input of the neurotransmitter serotonin from the dorsal raphe nucleus in the brainstem, which attenuates light-induced shifts in circadian phase (Rosenwasser, 2009). On the opposite end of the sleep-wake spectrum, sustained attention is critically dependent on the neurotransmitter acetylcholine projected from the basal forebrain to multiple areas of the cortex involved in sensorimotor and cognitive processing (Sarter et al., 2001). Although these mechanisms are often associated with the basic functions of sleep-wake rhythms, many hormones relevant to team behavioral health and performance (as described in subsequent sections) also exhibit natural circadian rhythms, including cortisol, testosterone, and oxytocin (Amico et al., 1983; Haus, 2007), which could systematically impact team dynamics based on scheduling as an organizational-level input to an IMOI team system. At the extreme end, circadian rhythm disturbances in sleep-wake patterns, hormones, and mood states are associated with, if not diagnostic of, psychiatric disorders including major depression, bipolar disorder, and schizophrenia (Cohrs, 2008; Vadnie and McClung, 2017; Pilz et al., 2018), which could profoundly impair team functioning and mission success in closed system ICE environments.

Under more conscious control are the sensorimotor systems largely responsible for the control, execution, and inhibition of motor behaviors. In a team mission context, this manifests in the overt physical performance of individual and team tasks and activities, and within the IMOI model could serve as an individual-level ability input potentially affecting mediating behaviors and team performance outcomes. These largely neuromuscular processes are regulated in the brain by the motor cortex, which projects to the basal ganglia in the midbrain, the brainstem, and spinal cord, terminating on motoneurons innervating muscles to execute movement (Lemon, 2008). Neural projections from the motor cortex largely discharge the excitatory neurotransmitter glutamate, with the basal ganglia and brainstem regions projecting the inhibitory neurotransmitter gamma-amino butyric acid (GABA), which disinhibits motoneurons, thereby allowing the release of acetylcholine to stimulate muscle activity (Sian et al., 1999; Grillner, 2015).

### Negative and Positive Valences

Moving to domains with more direct connections to behavioral health and social dynamics, the negative valence domain encompasses fear, anxiety, frustration, and loss. Within an IMOI context, variations in these systems may be considered abilities serving as individual-level inputs that contribute to the mediators of emergent team processes, affect, behaviors, and cognitions. Behavioral markers of fear, anxiety, and arousal include avoidance, social withdrawal, and characteristic facial and vocal expressions (or blunting thereof), whereas physiological outputs include increased heart rate, decreased heart rate variability, elevated and/or sustained levels of the hormone cortisol and neurotransmitters epinephrine and norepinephrine (defining features of the “fight or flight” stress response), increased inflammatory molecules [e.g., interleukins 1 and 6 (IL-1, IL-6), tumor necrosis factor alpha (TNF-α), C-reactive protein], and reduced nerve growth factors such as brain-derived neurotrophic factor (BDNF; Berntson et al., 1997; Phillips et al., 1998; Howren et al., 2009; Dowlati et al., 2010; Jaggar et al., 2019).

Critical to negative valence processes is the limbic system, a primitive set of structures seated deep within the brain that includes the bed nucleus of the stria terminalis (BNST), amygdala, and hippocampus (Lebow and Chen, 2016). Various clusters of cells (nuclei) within each structure receive and produce an array of neurochemicals that regulate projections to other structures and subsequent subjective, behavioral, and physiological responses to environmental and social stimuli. The BNST is subject to input from the neurotransmitters serotonin and dopamine, steroid hormones (estrogen and testosterone), and oxytocin and releases the inhibitory neurotransmitter GABA in projections to the hypothalamus. The amygdala is responsive to the excitatory neurotransmitter glutamate as well as estrogen hormones, opioid peptides, and oxytocin. Among other functions, the amygdala releases glutamate and corticotropin-releasing hormone (CRH) in projections to the hypothalamus (LeDoux, 2007; Myers and Greenwood-VanMeerveld, 2009). The amygdala, hippocampus, and hypothalamus all receive serotonin input from the dorsal raphe nucleus of the brainstem, with increased serotonin associated with the reallocation of energy and attention toward the precipitating aversive stimuli and reduced receptivity to positive stimuli (Andrews et al., 2015). Of particular relevance to ICE environments are connected with the hypothalamus, which is the leading point of the hypothalamic–pituitary–adrenal (HPA) axis of the biobehavioral stress system. Here, in anticipation of or response to a perceived threat or other excitation, CRH is released from the hypothalamus and binds to the pituitary gland, which releases andrenocorticotrophic hormone (ACTH). ACTH then enters and travels in the bloodstream until it binds to the adrenal glands to stimulate the release of cortisol and epinephrine; cortisol then returns to the hypothalamus in a negative feedback loop to dampen further activation (Pariante and Lightman, 2008). Although acute stress can provide transient boosts to physical and cognitive performance and immunity (Leonard, 2005), chronic stress and trauma can alter the structure and function of these mechanisms, with HPA axis dysregulation associated with myriad physical and neuropsychiatric conditions, including mood and anxiety disorders, cardiometabolic disease, post-traumatic stress, immune dysfunction, and dementia risk (Yehuda, 2001; Padgett and Glaser, 2003; Byers and Yaffe, 2011; Gianaros et al., 2015).

The negative valence domain and systems may dominate discussions of mission risk; however, the positive valence domain is no less relevant to social dynamics and team performance in ICE settings. Within an IMOI team model, the systems underlying positive valence could also be conceptualized as abilities serving as individual-level inputs, particularly critical to enable the reward and reinforcement processes necessary to build and sustain mediators of effective team processes and positive emergent states that feed into performance outcomes. Behavioral and physiological markers of positive valence are largely the reverse of those characterizing negative valence, i.e., approach behavior and social engagement, characteristic facial and vocal expressions, and reduced activation and/or persistence of physiological stress responses. Circuitry unique to the reward and reinforcement processes involves the mesolimbic reward pathway in the midbrain, featuring the ventral tegmental area (VTA) and nucleus accumbens (NAcc). The experience of pleasure, desire, and active pursuit of a wide variety of reinforcers (e.g., food, water, sex, social interaction, drugs, and art) includes GABA and glutamate input to the VTA, which projects dopamine to the NAcc. Dopamine release from the VTA to NAcc is a characteristic neurobiological definition of reward (Salamone et al., 2005); however, this circuit also connects to the negative valence systems, with inhibitory GABA projections to the BNST, amygdala, and hypothalamus (Salgado and Kaplitt, 2015). Structural and functional aberrations in the reward circuit, including decreased dopamine response to rewards and increased activation of the endogenous opioid system, are associated with anhedonia, addiction risk, social behavior deficits, and mood disorders (Nestler and Carlezon, 2006; Heller et al., 2009; Berridge and Kringelbach, 2015; Supekar et al., 2018).

### Cognition and Social Processes

The cognitive domain and associated mechanisms play a role throughout the team lifecycle, with key constructs including memory and cognitive control. The ability to acquire, retain, and recall learned knowledge, skills, and abilities is fundamental for individual and team functioning, particularly in high-performing teams operating in complex mission environments. Clearly, any moderating team cognitive processes such as shared mental models and transactive memory systems would depend on the integrity of the mechanisms enabling memory as individual-level inputs to an IMOI team system. Biologically, declarative memory (representations of facts, events, places, and people) is most associated with the hippocampus, which is part of the limbic system. Accordingly, its connections with the amygdala enable emotional input during encoding and emotional elicitation during recall/expression (Squire, 1992), with recall/reinstatement dependent on the neocortex (McClelland et al., 1995). Key neurochemicals underlying cognitive processing include acetylcholine, glutamate, epinephrine, opioid peptides, and GABA (McGaugh, 1992). A brain region especially relevant to cognitive control and virtually all neurobehavioral domains is the prefrontal cortex (PFC; Fuster, 2001). Evolutionarily speaking, it is a relatively new structure compared to the limbic, midbrain, and brainstem regions and is especially prominent in humans. The PFC receives and integrates input from all sensory and motor regions, as well as the limbic system (Miller and Cohen, 2001). The PFC also projects throughout the brain, including extensive interactions with the hippocampus in the processing and recall of both recent and remote memories and excitatory glutamate projections from the orbitofrontal region of the PFC to the NAcc in reward processing (Lynch, 2004; Frankland and Bontempi, 2005). The PFC is largely known for its role in integrating information and regulating executive function required for judgment and decision making, abstract reasoning and concept formation, and planning for the future and is a major source of inhibitory control throughout the brain. Recent work relevant to both the negative valence and social process domains suggests a relationship between decreased structural and functional integrity of the right orbitofrontal, left dorsolateral, and anterior cingulate cortex regions of the PFC and increased antisocial, violent, and psychopathic behavior (Yang and Raine, 2009), any of which could constitute a critical threat to mission success in ICE operations.

Finally, the social processes domain is clearly related to social dynamics and team performance, with its key constructs of affiliation/attachment and communication. Within an IMOI system, receptivity and capacity for affiliation and effective communication are core skills and abilities for individual team members in the mixed work/social setting of long-duration missions in ICE environments (Landon et al., 2017, 2018; Roma and Bedwell, 2017) and serve as essential individual- and team-level inputs to virtually all mediating team processes, emergent states, and behaviors. Biologically, perhaps the best-known mechanism involved in social processes is the hormone oxytocin. Oxytocin in the brain is produced by cells in the hypothalamus (Lemos, 2012), released *via* the posterior pituitary gland, and binds to receptors in the BNST, amygdala, NAcc, and hippocampus (Boccia et al., 2013). Acute oxytocin reportedly increases gaze to the eye region of human faces, increases trust, improves the ability to infer emotional states in others from facial cues, and enhances the stress-reducing effects of social support (Heinrichs et al., 2003; Ross and Young, 2009), presumably through reduction in social anxiety enabled by the aforementioned projections to the limbic system (Feldman, 2012). However, oxytocin and the social affiliation it enables are not always positive, as oxytocin can strengthen in-group bonds at the expense of out-group relationships, including increased deception and ethnocentrism toward those perceived as “others” (Bartz et al., 2011; De Dreu et al., 2011; Eckstein et al., 2014; Shalvi and De Dreu, 2014). In addition to oxytocin, gonadal hormones progesterone and testosterone are also relevant to social cognition and processes. Although these hormones are produced outside the brain, they can easily pass the blood-brain-barrier and bind to structures such as the BNST, amygdala, hypothalamus, and NAcc. Despite their traditional association with reproductive behaviors, mood, and aggression, recent evidence also suggests that these hormones play a moderating role in human social dynamics, group stability maintenance, and team effectiveness. Specifically, higher progesterone is associated with lower emotion recognition and stronger affective responses to faces (Derntl et al., 2013), whereas higher testosterone is associated with increased fairness behaviors, higher social status, and social inclusion (Edwards et al., 2006; Eisenegger et al., 2010, 2011; Seidel et al., 2013; although see Zyphur et al., 2009).

Taken together, even with this intentionally limited and simplified review of key neurobehavioral domains relevant to individual and team behavioral health and performance in ICE environments, it should be clear that the brain is an extraordinarily complex system unto itself. Indeed, this multileveled and interactive complexity is in part what enables humans to adapt to such a wide variety of physical, social, and environmental demands. However, the complexity and interconnectedness of these neurobiological systems also make them subject to modification by those very same demands, especially in ICE settings. The following sections describe the importance of several critical components of ICE systems outside the traditional team science disciplines, and how those factors may act on our core neurobehavioral systems to affect and be affected by social dynamics in ICE environments over time.

## Multidisciplinary Contributions to Isolated, Confined, and Extreme Teams

### Food and Nutrition

#### Overview of Food and Nutrition

Any operational environment in which people live must include a food system. In addition to the obvious necessity of food to sustain life, the food system has two core roles in supporting human psychosocial health. First, adequate intake, absorption, and utilization of specific nutrients are essential to promote behavioral health and cognitive function on a biochemical level directly or through influence of the gut microbiome. Second, food has a social role as a shared activity, providing a familiar comfort for mealtime gatherings that may become increasingly important in isolation and confinement where other comforts and reminders of home are not available. Food variety, availability, quality, nutrient stability, ease of preparation, dining accommodations, and timing of meals all impact adequate food and nutritional intake and associated behavioral health and social cohesion, as reported previously in reviews of food systems in military, spaceflight, and historic exploration settings (Marriott, 1995; Stuster, 1996, 2016; Douglas et al., 2016).

Space food to date has been processed and individually packaged to support multi-year shelf stability and ease of preparation. Refrigeration is not available for foods on the International Space Station (ISS), with extremely limited availability of fresh produce only when a resupply vehicle docks, which will likely not be available during exploration class missions to Mars. Astronauts on the ISS consume a standard menu and only receive a small selection of shelf-stable personal preference items; therefore, it is restricted in both quantity and variety. Customization of space foods from the standard menu is limited to the addition of condiments and selection of foods within the standard menu food containers. Crew members are not required to consume a specific menu each day, but they are constrained by availability of foods and their crew mates’ likes and dislikes. For example, if a crew member likes one specific food item, that food item will only appear in the standard food containers 2–3 times every 7–9 days. Crews are permitted to open a new set of standard menu food containers every 7–9 days, depending on the caloric requirements of the crew during each mission (Douglas et al., 2016).

#### Nutrition and Social/Team Factors

##### Specific Nutrients That Affect Individual Mood and Behavior

Nutritional deficits can affect the pathophysiology of mood disorders including depression, which can in turn affect individual performance within a team, healthy, and constructive team interactions, and may cause the withdrawal of that individual from team activities. Zinc deficiency is one example of an essential nutrient for maintenance of normal brain function and has been associated with increased depressive-like and anxiety-related behavior (Roohani et al., 2013; Mitsuya et al., 2015). In addition, low vitamin D status and insufficient omega-3 fatty acids are others that are associated with mood disorders because of their link with the production and action of serotonin, a neurochemical that is typically lower in major and bipolar depression, schizophrenia, and other mood disorders (Patrick and Ames, 2015). Not only do vitamin D receptors exist in the brain, but also low vitamin D status has been shown to negatively affect neural activity and cellular activity in the brain (McCann and Ames, 2008). A higher vitamin D status (serum 25-hydroxyvitamin D) has been demonstrated to significantly reduce risk for depression (Ju et al., 2013); however, vitamin D supplementation studies that have looked at effects on depression have mixed results. Vitamin D has a more profound effect on mitigating symptoms in cases of more severe depression and lower vitamin D status (Shaffer et al., 2014). Several epidemiological studies have found inverse correlations between oily fish consumption and bipolar or depressive symptoms (Grosso et al., 2014).

With increased ionizing radiation exposure on deep space exploration missions, blood-brain barrier function needs to be considered for nutrients that are concentrated in the brain *via* active transport processes. One example is the B-vitamin folate. A compromised blood-brain barrier due to chronic low-dose ionizing radiation exposure or other factors could lead to cerebral folate insufficiency, which has been associated with many neuropsychiatric disorders including depression and schizophrenia (Molero-Luis et al., 2015).

Not only does nutrient intake directly affect nutrient status and behavioral health, but also the nutritional adequacy of the diet is a prime influence on the composition of the gastrointestinal (GI) microbiome (David et al., 2014). GI microbes metabolize available components of the diet, including those indigestible to humans (e.g., fiber and flavonoids not absorbed in the small intestine from fruits and vegetables), into short chain fatty acids, peptides, phenolic acids, and neurotransmitters that may impact social behavior, memory, and cognition through the gut-brain axis (Stilling et al., 2014; Dinan and Cryan, 2017; Vuong et al., 2017; Tengeler et al., 2018). For instance, some *Lactobacillus* species used in food fermentations are capable of producing GABA (Barrett et al., 2012; Ribeiro et al., 2018), which may be associated with reduced anxiety and depression through its actions on the negative valence mechanisms described above (Lydiard, 2003). The GI microbiome has also been suggested to impact production of neurotransmitters such as serotonin, or its precursor, tryptophan (Desbonnet et al., 2008; Wikoff et al., 2009; Wall et al., 2014). Dietary factors, including fat, fiber, flavonoid, and sugar content of the diet can also influence microbiome diversity. Flavonoid compounds in plants can impact specific strains of bacteria by inhibiting growth of some or promoting growth of others (Nohynek et al., 2006; Xie et al., 2015). Generally, a high fat, low fiber, and high sugar diet decreases bacterial diversity and increases inflammatory processes contributing to metabolic syndrome, insulin resistance, and neuro-inflammation and behavioral disorders (Kim and de La Serre, 2018). Conversely, lower fat, high fiber diets contribute to increased bacterial diversity, decreased inflammation, and strengthening of the gut barrier. There are a number of spaceflight factors that still have unknown effects on the GI microbiome, including the processed food system with a high quantity of sterile foods, and radiation exposure, but it is clear from ground-based research in humans and animals that the microbiome can affect cognitive function and behavior.

Microorganisms with probiotic psychiatric effects, meaning they can produce a health benefit if consumed in adequate amounts, have been described as “psychobiotics” (Dinan et al., 2013). Evidence from both animal studies and human clinical trials supports that ingestion of psychobiotics, many of which are associated with foods and supplements, can reduce symptoms of stress, anxiety, and depression (Stilling et al., 2014; Sampson and Mazmanian, 2015; Douglas and Voorhies, 2017). The GI microbiome may also influence the brain, mood, and behavior through interaction with the immune system (Rothhammer et al., 2018; Sylvia and Demas, 2018) or through production of odorants that act as social cues (Bienenstock et al., 2018). Although human studies in these areas are limited, a preliminary investigation in a confined 105-day human analog study indicated a potential relationship between GI microbial composition and mood (Li et al., 2016). Considering the substantial impact that the GI microbiome may have on cognitive function, neuro-inflammation, and behavior, the impacts of the spaceflight diet, crew food selection, and environment on the GI composition warrant further investigation.

##### Connections of Food/Nutrition to Team/Social Behaviors

Even with the limitations in the food systems in ICE environments, food is often identified in ISS astronaut debriefs as one of, if not the most, important factors to morale (Douglas et al., 2016). Food was within the 10 most discussed categories identified in an analysis of astronaut journals, both as a source of frustration and as a source of pleasure depending on factors such as the variety, availability (resupply), and quality of chosen items and the adequacy of the space available for group meals (Stuster, 2016). Allowing crew members to self-select what food items they want to consume each day (within the food containers that are opened at that time) yields greater crew satisfaction as documented in ground analogs using closed food systems for extended periods of time (Milon et al., 1996). The European mission simulation study EXEMSI (60-day confinement) results clearly demonstrated that specific menus should not be imposed on the crew, but menu suggestions should be available. They note that in an environment with multiple stressors, food should not be considered as an additional stressor but should allow for personal choices.

The limited quantity and variety of foods in ICE settings can be a potential source of contention. This was demonstrated in the Mars 500 analog, where lack of culturally acceptable variety and differences in cultural eating habits may have cause friction among crew members (Šolcová et al., 2016). It was recommended that more attention should be focused on the design of the food system (nutrition, variety, multicultural expectations, etc.) to prevent issues in future missions. However, food also was one of the most discussed topics and acted as an important natural bridge for the multicultural crew.

The importance of food and group meal times to team cohesion is evident in human exploration accounts (Stuster, 1996, 2016). Exploration researchers have recommended that the entire crew eat together regularly to support communication and prevent subgroup formation (Stuster, 1996). Timing is an important consideration to group meals, and food rehydration and heating equipment on NASA spacecraft must be designed to support simultaneous food preparation and group meals even when schedules are demanding. Even though Skylab was the only space program with high-quality refrigerated and frozen foods, time pressure in relation to meal preparation reportedly reduced the number of group meals (Stuster, 1996). Over the course of a mission, special meals that occur on a predefined basis and celebratory meals have been noted to help mark the passage of time.

Crew self-selection of food items within the limited food system, rather than adherence to a guided menu, can also unintentionally affect nutrient status and resulting behavioral health among individuals. There are examples of chamber studies with closed or semi-closed food systems where crew members did not get enough nutrients through the food system even though the planned food system contained enough of each nutrient. One example where a 60-d closed food system provided nutrient requirements but actual vitamin intake (particularly vitamins B1 and B6) was below the dietary requirements is the European Space Agency’s ESA EXEMSI study, which indicates that the crew members were not selecting completely nutritionally balanced meals (Milon et al., 1996). Another example is from Biosphere 2, where a crew of eight lived in an environment with finite natural resources for 2 years. In this system, vitamins D and B12 were deficient according to government RDA standards (Silverstone, 1997). A 105-day chamber study in Russia also showed that crew members who intentionally excluded specific food items, such as protein-rich desserts, became protein deficient and lost body mass (Agureev et al., 2017). These examples underline the importance of food selection and crew preferences in preventing deficiencies in nutrients that can in turn affect behavioral or cognitive health.

The impacts of a limited food system on social and team behaviors may be more severe in future long-duration exploration missions. The food may be sent multiple years ahead of a mission and selection of the crew and therefore limited to a standard menu devoid of individually selected preference foods or fresh foods. If a crew member chooses to eat only limited types of foods from this system, it may cause conflict by unacceptably restricting the availability of those foods for others. Additionally, if a member of the crew limits their food choices from an allotment of food that has been prepositioned on a lunar or planetary surface, it may prevent the intake of a balanced diet for all crew members and could result in nutritional deficiency and potential downstream effects on physical and behavioral health and performance. Of greater concern to team cohesion would be dishonorable food practices. An incident of food being “plundered” was mentioned in an ISS astronaut journal, which served as an acute social stressor producing feelings of resentment (Stuster, 2010).

The direct introduction of chemicals to the body *via* the nutrients in food is just one component of ICE systems that can directly impact the neurobiological systems underlying adaptation and social dynamics in ICE settings. Invoking the body’s physiological systems as work, play, or maintenance activities is another inherent component of ICE systems that can directly alter physiology and impact the key neurobiological systems affecting physical readiness to perform team tasks and cognitively engage in social behaviors.

### Exercise and Physical Activity

#### Overview of Exercise Physiology

In spaceflight, the risk of decreased musculoskeletal health and cardiorespiratory fitness is largely driven by microgravity. In microgravity, humans do not experience continuous daily loads on the body as they would in Earth’s gravity, and as a result, bone and muscle tissue weaken. This deconditioning poses danger upon return to Earth and for future missions to the moon and Mars, which may involve planetary surface operations under corresponding gravity-related loads. Exercise is a critical countermeasure to prevent multi-system deconditioning during spaceflight and should also be used to target mitigation of the stressors associated with spaceflight (i.e., isolation, confinement, and other stressors) to promote team cohesion and mission success. Exercise devices in space have improved significantly since the early decades of spaceflight, and current countermeasures onboard the International Space Station (ISS) include a treadmill with a restraining harness and Advanced Resistive Exercise Device (ARED), allowing for cardio and load-bearing workouts for long-duration crew members (Ploutz-Snyder et al., 2015). Similar to military and firefighter physical fitness requirements and guidelines for other physically demanding jobs, crews must maintain adequate physical fitness for their missions. To this end, crew members are scheduled for exercise 6 days a week, for up to two and a half hours per day in-flight.

The favorable effects of regular exercise on multiple physiological systems and psychological health dates back to teachings from Confucius and ancient Greek philosophers who recognized exercise and physical fitness as essential factors to maintain health, strength, and a prolonged life (Berryman, 2010). Current literature has indisputably shown the benefits of regular exercise across multiple domains, including treatment for depression (Cooney et al., 2014), motor skill acquisition (Roig et al., 2012; Statton et al., 2015), cognitive function (Chang et al., 2012), and sleep quality (Reid et al., 2010). Within operational environments, exercise can be used not only as a countermeasure to maintain muscle strength and cardiovascular fitness but also as a critical mediator of stress responses to promote physical and psychological resilience. Regular physical activity buffers against depression and anxiety, and greater calmness, better mood, lower anxiety, and a generally lower susceptibility to life stressors have been shown in trained individuals compared to their less fit counterparts (Silverman and Deuster, 2014). In addition to improving these factors, physically fit individuals experience significantly less stress compared to unfit individuals during physical activity at the same work rate as demonstrated by lower heart rate responses and cortisol levels (Deuster and Silverman, 2013). From the perspective of promoting resilience, studies have demonstrated that self-esteem and self-efficacy are improved through regular physical activity (Delignières et al., 1994; McMurray et al., 2008).

More recently, the state of knowledge on the effects of exercise on neurobiology has expanded and allowed for more detailed understanding of *how* exercise promotes factors such as resilience, stress tolerance, and adherence to exercise. Exercise directly enhances brain function by regulating peripheral and central nervous system growth factors including brain-derived neurotrophic factor (BDNF), insulin-like growth factor 1 (IGF-I), and vascular endothelial-derived growth factor (VEGF). Exercise-induced increases of BDNF and IGF-1 can improve learning and reduce depressive symptoms through supporting the growth and repair of blood vessels and brain tissue that support overall cognitive functioning (Cotman et al., 2007; Silverman and Deuster, 2014). Emerging work suggests that the hormone osteocalcin, which is produced exclusively in bones and maintained or increased with exercise, can act on the brain and may mitigate anxiety and cognitive deficits (Obri et al., 2018; Shan et al., 2019); this is particularly relevant to teams in space, where exposure to the microgravity environment can reduce osteocalcin levels without sufficient exercise (Smith et al., 1999; Garrett-Bakelman et al., 2019). Thus, exercise can directly help support the mechanisms underlying the knowledge, skills, and abilities necessary to sustain team processes and performance throughout a mission.

#### Exercise and Social/Team Factors

Exercise provides a unique countermeasure to enhance brain health and function by indirectly reducing the peripheral risk factors associated with cognitive decline and directly enhancing the brain health and cognitive function. As described above, the stress response is regulated by the HPA axis, autonomic nervous system, and immune system. Activation of these systems causes release of cortisol and epinephrine to enable the response of other body systems (cardiovascular, musculoskeletal, nervous, and immune) to meet the demands of the challenge presented and then return the body back to normal levels. Importantly, timely termination of the stress response is critical for preventing systemic inflammation, which is detrimental to physical and psychological health over time. Maintaining physical fitness effectively reduces constant systemic inflammation by quickly returning chemicals released during a stress response to baseline levels (Silverman and Deuster, 2014).

Studies addressing the effects of exercise on psychological health usually focus on the individual; however, in the context of ICE environments, it is critical to also explore how exercise can improve team cohesion and performance. Most mission activities performed during spaceflight missions require crew members to work together, and even if it is not a requirement, activities can typically be completed more efficiently and effectively with the help of crewmates. Extravehicular activity (EVA), colloquially known as a “spacewalk” among astronauts, and other mission-critical team tasks are one of the most important team activities performed on missions and exemplify the need for ICE teams to perform with high levels of team cohesion and cognitive functioning in a high stress environment. Every step of an EVA from training to preparation to return to the vehicle is well-planned and practiced. It requires all crew members to perform their individual tasks well, has situational awareness of each other’s well-being and location, effectively communicates with each other and ground support, and offers supporting behaviors to assist each other. Even with optimal preparation, unexpected events occur during EVAs that require the crew members to work together toward a solution. In these cases, it is critical that EVA crew members possess self-efficacy and execute team processes such as collaborative decision making and backup behaviors. Additionally, EVAs are typically 6 or more hours in length and are very physically and cognitively demanding. Fatigue may cause cognitive errors to increase and communication to decrease, so exercise to build endurance for these events is essential. As we progress to future planetary exploration EVAs, especially during longer duration missions, EVAs are likely to be less tightly scripted, and therefore, team cohesion and good team process become even more important as the team must work autonomously to address dynamic challenges.

The most effective combinations of exercise volume, intensity, and modality to promote psychological health are not known and likely vary between individuals. Most studies in this area have focused on cardiovascular-based exercise rather than resistance exercise training. It appears that moderate to vigorous intensity aerobic exercise is the most effective (Chang et al., 2012), likely due to the fact that the cascade of catecholamine and growth factor responses is minimal with lower intensity exercise. The effects of resistance exercise on brain health are less studied; however, preliminary evidence suggests that higher load, low repetition resistance exercise stimulates areas of the brain differently than lower load, higher repletion exercise (Kraemer et al., 2013). Understanding the molecular and brain area specific responses associated with different exercise and physical activity profiles during spaceflight and other ICE mission settings will be critical in optimizing exercise hardware, software, and prescriptions for maintaining physical and behavioral health and performance capacity for individuals and teams in extreme mission operations.

### Sleep/Wake/Work Rhythms

#### Overview of Sleep and Fatigue

ICE operational environments often include irregular or unnatural work schedules, light/dark cycles, and sleeping environments. For example, Antarctic researchers and submariners may not see the sun for months, while astronauts in low Earth orbit see a sunrise or a sunset every 45 min. Excerpts from astronaut journals collected during spaceflight missions have identified fatigue and sleep as a major source of stress and relief, mentioned hundreds of times (e.g., Stuster, 2010, 2016). In contrast to pure muscle fatigue, mental fatigue is the “inability to function at one’s optimum level, because physical and mental exertion (of all waking activities, not only work) exceeds existing capacity” (Gander et al., 2007). Sleep is a necessary biological process that allows the brain and body to recover from the day’s scheduled and unscheduled physical, cognitive, and social activities. Humans on average prefer approximately 8–8.5 h of sleep per night to maintain health and cognitive functioning (Klerman and Dijk, 2005). Notably, astronauts often do not receive a full night’s sleep while on a mission, instead averaging 6 h of sleep per night, due to the physical and psychological stressors inherent in an operational mission (Barger et al., 2014). Sleep supports many physiological processes such as maintaining muscle, organ, and immune functioning and encourages repair and restoration through the release of chemicals such as growth hormone (Kim et al., 2015). During sleep, cerebrospinal fluid within the brain flushes out waste products of cell functioning that accumulates during waking hours, effectively cleaning the brain (Xie et al., 2013). Sleep also supports memory consolidation. Outside influences may cause fatigue such as the sleep environment, the time of day and circadian rhythm, quantity and quality of sleep, and total or partial sleep deprivation. Sleep environments that are too hot/cold, noisy, bright, and prevent reclined positions reduce sleep duration and may lead to more awakenings. Relying on sleep during typical times of alertness, or working during hours typically reserved for sleep (e.g., pulling an “all-nighter”), results in poor quality and insufficient sleep. Sleep loss may be both an acute issue and a chronic issue; that is, sleep deprivation may come in the form of missing all or part of a typical night’s sleep, or a reduction in sleep duration for a period of several nights. Both acute and chronic sleep restrictions negatively affect individual performance and well-being (Cohen et al., 2010).

There are also several factors that may influence individual sleep and fatigue patterns. Studies have found that individual sleep needs and preferences as well as the response to sleep loss and fatigue vary according to genotype (Groeger et al., 2008; Vandewalle et al., 2009). These differences in the underlying genotypes may drive affect, behaviors, and cognition. For example, variants in the PER3 gene expressed in the suprachiasmatic nucleus (SCN) of the hypothalamus that regulates sleep and circadian rhythms have been associated with the differential activation of the parietal and temporal lobes of the brain under conditions of sleep loss, resulting in poorer performance (Vandewalle et al., 2009). In other words, some individuals are more vulnerable to the effects of fatigue and require more recovery from fatigue than others. These and other influences of fatigue are well documented in the literature, as are the outcomes in the multiple neurobehavioral domains. As a brief list of common outcomes, fatigue has been linked to affective decrements in emotional stability, self-regulation, positive affect, and motivation; behavioral outcomes of reduced physical activity, less accurate assessment of risk, and less and poorer quality communication; and cognitive outcomes of cognitive slowing, reduced attention and recall, poor decision making, and increased risk of errors (Chabal et al., 2018; Banks et al., 2019). When placing these findings in a team context, individual differences in reactions to sleep loss, work overload, and schedule shifting can impact each team member in a unique way, introducing variability in performance and social functioning that must be addressed by the team.

#### Fatigue and Social/Team Factors

Sleep need and vulnerability to fatigue are primarily individual-level input variables in the IMOI model. Differences related to fatigue vulnerability, and an individual’s chronotype (i.e., whether the individual is a morning lark or night owl) stems from endogenous individual differences and general physiological health. However, these individual-level inputs may directly influence patterns of interacting with team members. For example, in the Mars-520 mission simulation analog study, one of the six crew members was a habitual napper, which reduced their interactions with other crew members by 20%, while another crew member developed a free-running sleep-wake schedule in which his circadian rhythm (and thus, regular interactions) became misaligned with all other crew members (Basner et al., 2013). These crew members’ asynchrony effectively reduced the crew’s collective knowledge and skills, altered the team structure and team size, and reduced the manpower for team processes such as systems and goal monitoring, backup behaviors, and coordination. Communication, an essential component of teamwork, is decremented at the individual level under conditions of fatigue. The few team studies of fatigue and communication, conducted most frequently in military populations, found teams either reduced or stopped communications, which decreased performance, and sought more visual forms of information (Whitmore et al., 2008; Fletcher et al., 2012).

In a closed environment such as a long-duration space expedition or a deployed military submarine team, team members function as both coworkers and roommates. Spending less time together due to misaligned sleep/wake/work schedules may not only affect team task cohesion (i.e., working well together toward a goal) but also influence team social cohesion (i.e., shared attachment and liking) through reduced time spent sharing meals, engaged in recreational activities, or being available to provide and receive social support. A reduction in time spent together, particularly as it may be expressed differently among circadian misaligned team members, may create fractures within the team. As team cohesion has been positively linked to team performance (Mathieu et al., 2015), reduced social support and team cohesion related to circadian misalignments may result in poor team outcomes. The cohesion-performance relationship has also been found to be reciprocal in studies of isolated teams in Antarctica and mission simulations (Kozlowski et al., 2015). Thus, reduced team cohesion begets poor performance, which further reduces cohesion, and fatigue acts as an amplifier of this downward spiral. Other affective states of team confidence and trust may also suffer as a fatigued team member is more likely to demonstrate emotional instability, commit cognitive lapses, or withdraw from the team altogether. Identification of others’ needs for social and emotional support may also be neglected as sleep-deprived individuals are less able to recognize facial displays of human emotions (van der Helm et al., 2010). Over time, teams that are not able to rely on the regular presence, consistent performance and support, and emotional stability of all team members are likely to see a reduction in team performance and team functioning that accumulates as this negative pattern persists. Consequent issues related to poor team performance may also negatively influence each individual team members’ ability to sleep as they ruminate on negative team situations and performance outcomes. The level of fatigue, either driven by psychological reactions to a team situation or by physical needs (e.g., staying awake 36 h to address an emergency), becomes inputs for the next cycle of the IMOI, influencing the team through each individual’s vulnerability to the new level of fatigue. Notably, the team may be able to compensate for the fatigued individual in such a way that they avoid the decrement to performance. For example, a laboratory study of team decision making found errors increased at the individual level, but these effects were attenuated by team membership (Baranski et al., 2007). We currently do not know what degree of fatigue within each team member and across the team is the tipping point for the decline in performance and functioning. Determining this threshold, particularly for small teams in a high-risk ICE operational environments with irregular work schedules or non-Earth-like light/dark cycles, would allow optimization of mission planning and timely deployment of interventions to support individual and team behavioral health and performance.

### Habitability and Systems Design

#### Overview of Habitability and Human Factors Design

By its nature, human occupation of extreme environments requires specially designed habitats and equipment to allow operational teams to achieve their mission objectives and maintain safety. Indeed, the “extreme” portion of ICE typically refers to a dangerous external geophysical environment incompatible with human physiology, health, and well-being, including the lack of or toxic atmosphere, extreme altitude (above or below sea level), extreme heat or cold (or rapid shifts between the two), non-24 h light-dark cycles, reduced gravity, wildlife threats (e.g., predatory animals, microorganisms, toxins), or potential exposure to radiation and extreme weather phenomena (e.g., solar flares, high winds, dust storms, rough seas, blizzards, and volcanism). An extreme level of even necessary isolation brought about by physical constraints, physical confinement, austere environmental conditions with little to no natural sensory stimulation, and social loss due to the inability to communicate with others outside the immediate team in real time all have the potential to impact both individual and team function. A habitat that not only protects from physical external threats but supports individual health and performance and facilitates positive team dynamics must be carefully designed. A poorly designed habitat can negatively impact crew members by inducing acute and chronic stress responses in the individuals living and working in the operational environment. These effects may be magnified under increased mission duration and isolation and could constitute a chronic stressor (Celentano et al., 1963; Mohanty et al., 2006). Several features of habitats that are important to team function in situations of extreme isolation and confinement are discussed below. Essentially, the habitat should enable effective performance while accommodating group activities and providing sufficient privacy and means of escape from the mixed work/social setting of closed ICE mission environments.

#### Habitability, Human Factors, and Social/Team Factors

##### Group Activities

ICE habitats should allow for a crew to gather together within the same space for not only work functions but also recreational opportunities. As discussed by Ozbay et al. (2007), low social support has been associated with physiological and neuroendocrine indices of heightened stress reactivity, including elevated heart rate, increased blood pressure, and heightened cardiovascular and neuroendocrine responses to stressors. Habitats designed for long-duration missions should ensure adequate physical space to facilitate social support.

One of the major contributors to interpersonal conflict highlighted in polar and spaceflight expeditions is the tendency for the formation of subgroups within the crew (Stuster, 1996). Providing an environment that supports group communication may mitigate this issue and lead to a more cohesive team (Bender and Fracchia, 1971). As mentioned, Stuster suggested that meals may offer this type of communication and social support opportunity, where the entire crew can gather to prepare their food and dine together. Consequently, it is important to provide a space in the habitat that allows for this type of casual group interaction. Evidence from a study of ISS astronaut journals emphasizes the need for this space to facilitate group communication and enhance team cohesion (Stuster, 2016).

Evidence for the importance of dining together led to the creation of a NASA Human-System Standard (NASA, 2015), which states that crew members shall have this capability to support crew psychological health and well-being (NASA Standards 7.1.2.5 Dining Accommodations). The standards provide a baseline for future spaceflight programs, which design vehicle habitats with consideration to crew health within mission resource limitations and mission length and distance. This entails consideration for sufficient physical volume and designs the mission timeline and food system (e.g., ability to prepare meals at the same time) to support team meals. The Standard serves as one clear example to highlight the importance NASA places on allowing the crew to share physical space to support team cohesion. The design of the common galley area should also be considered, which should include a table that accommodates the entire crew without inadvertently creating tension. For example, Raymond Loewy, a “Habitability Consultant” for the Saturn-Apollo and Skylab programs, had a triangular table installed in the Skylab wardroom, so that “no man from the three-person crew could be at its head” (Mohanty et al., 2006). In many cases, the galley where crew members gather to share meals can also provide sufficient volume for other group recreation as well as work-related team tasks. Indeed, the importance of recreation to psychological health and well-being has been researched extensively. In the context of space exploration, both individual (e.g., reading) and group (e.g., watching a movie) recreation opportunities should be provided. The habitat should therefore accommodate both types of stress-reducing recreational activities.

For work-related team tasks, the galley or other areas designed to accommodate multiple crew members should carefully consider the nature of the team task as it relates to noise interfering with communication, physical or sensory interference of each person performing their duties in concert with the other, and whether the location of the team task blocks access to other important areas (e.g., sleep quarters), which may cause team frictions and frustrations, and negatively influence performance and efficiency (Kearney, 2016). Other critical factors to ensure teams are able to share information, foster trust, and coordinate efficiently include allowing common spaces for communication (e.g., digital whiteboards and shared displays), physical layouts that allow for eye contact and mutual viewing, and norms and standards for common labeling, stowage locations of tools and equipment, and adequate work spaces.

##### Privacy

While it is important to ensure that the volume and layout of a habitat facilitates team cohesion and performance through shared spaces, purposely private spaces for each crew member should also be provided, particularly in vehicles intended to support longer duration missions. Terrestrial studies have demonstrated that the experience of privacy—that is, *privacy* as a dynamic and dialectic interaction with others, whereby privacy represents the level of selective control one has over sharing one’s self with others (Altman, 1977)—is related to the architecture of privacy (Laurence et al., 2013), such as the design of a workspace with four walls. Hence, architectural private spaces facilitate the experience of privacy, which has been shown to be related to improved work performance (Karlin et al., 1979; DeCroon et al., 2005). The provision of a private space also allows for withdrawal from increased social density. In an assessment of social density and perceived control in high density residential neighborhoods, individuals living in areas with stores (compared to individuals living in residential areas without stores) reported more crowding, less ability to regulate social interactions, and lower perceptions of control (Fleming et al., 1987). In addition, they evidenced higher stress levels, including more somatic and emotional distress, and elevated urinary epinephrine, norepinephrine, and dopamine.

One exploration researcher contends that the majority of interpersonal conflicts arise from relatively minor issues that become exacerbated due to the extreme isolation and inability to escape one’s crewmates (Stuster, 2010). He asserts that the constant interpersonal interaction caused by a confined environment is a source of stimulation (and exacerbated by a smaller crew), and people need to occasionally withdraw from this stimulation in order to cope with the stressors of the mission and environment. The habitat should facilitate the individual crew members’ ability to withdraw from the rest of the crew, in order to conduct solitary activities. If no specific area is provided for privacy, crew members will likely improvise and modify their environments in order to achieve some privacy. Notably, these consequences are likely to accrue in the continued absence of privacy. The ability to withdraw and have physical (auditory and visual) privacy can help mitigate interpersonal conflict and support team health and performance.

The provision of an individual sleeping quarter has been the subject of debate for years. The Risk and Management Team of NASA’s Human Exploration and Operations Mission Directorate (HEOMD) published a report detailing lessons learned from the ISS program and recommendations for future exploration programs (Lengyel and Newman, 2014). Among these recommendations, the suggestion is that “crew comfort and privacy must be ‘front and center’” for spacecraft designed for long-duration space missions and recommends that future exploration vehicles provide crew member with a private sleep quarter, despite the engineering constraints on volume and habitat size. The authors cite feedback from crew members about the importance of having a private sleep quarter they can personalize and use for privacy. Evidence from ISS crew debriefs indicates sleep quarters that are valued and necessary spaces for conducting personal activities, and crew members emphasize the psychological benefit of having these private accommodations (Whitmore et al., 2013). Crew members also noted the importance of having the ability to decorate and personalize their private crew quarters (Kearney, 2016). Evidence for the benefit of providing crew members with a private sleeping quarter for long-duration missions has also been captured by NASA Standard 7.9.2 Private Quarters, which states that private quarters shall be provided to support crew health and performance for missions longer than 30 days. Whether or not an individual sleeping quarter is provided per crew member, the ability to retreat and achieve privacy from the rest of the crew members should be provided by the habitat. Both visual and auditory privacy should be considered in the design of private spaces. Chronic stress due to reduced privacy and increased social density of such environments may be further compounded by acute stress events related to habitability (e.g., temporary damage to part of the habitat reducing overall net habitable volume and increasing crowding for a short time). More generally, chronic and acute stressors related to habitability may interact with stressors related to any of the other topic areas we have discussed in this article, resulting in a continuous threat to the behavioral health, performance, and effectiveness of the crew.

## Opportunities for Research and Application

Examining the interaction of these seemingly disparate research areas of biology with team research is overdue, but there are several specific gaps in the literature that may serve as starting points. Uniting each of these areas should be a focus on the brain. That is, identifying the complex chemical interactions and neurobiological mechanisms influenced by nutrition, exercise, fatigue, habitability, and interactions with other individuals should acknowledge the potentially compounding effects of other areas in research designs. The resulting social and team behaviors of this interplay have received some targeted attention (e.g., studying the influence of one particular molecule on mood or tendency to withdraw from the team), but simultaneous consideration of multiple influencers on the brain is the next step. Throughout this review, we integrated several frameworks, including the IMOI model of team performance, the NIMH RDoC framework for basic neurobehavioral functioning across multiple levels of analysis, and the unique characteristics of ICE environment contexts. Ultimately, if understanding and enhancing team performance and social dynamics are the priority, then we believe that the IMOI framework is capable of serving as a guiding framework for research and development in the behavioral biology of extreme teams. Indeed, the IMOI model is not rooted in team performance but is rather an adaptation of systems theory and modeling. We consider our expansion of the individual input level in the IMOI model to include biologically relevant variables not so much a radical departure from organizational theory than a more realistic (albeit complicated) consideration of factors acting on the brain to affect individual and team behavioral health and performance over time. Characterizing these interrelationships and developing evidenced-based best practices and countermeasures is the exciting challenge facing the applied research community.

For nutrition, physical outcomes of inflammation and changes to the gut microbiome influenced by diet may also influence individual stress and physical and cognitive readiness to perform on the team. Research into providing adequate nutrition to sustain brain and body functioning with limited resources in a closed system should seek to understand potential affective, behavior, and cognitive effects of specific nutrients and foods. Researchers must also inform dietary countermeasures by understanding optimal methods for encouraging continued consumption of nutritious foods with a likely restricted variety, perhaps by leveraging social influence, team processes, and reward circuitry. Examining the social importance of shared meals for encouraging consumption, bonding as a team, and fulfilling social support and relaxation needs is a multifaceted issue naturally suited to a multidisciplinary approach incorporating biological, behavioral, cognitive, and social factors.

These issues are also applicable to exercise physiology research, which similarly investigates sustaining motivation to exercise over time, the benefits of group and competitive exercise, the use of exercise to reduce stress, and other psychological benefits to maintaining physical readiness and brain health to perform in a team. However, much of the data reported in these fields are based on study populations not representative of astronauts or other high-performing teams in long-duration extreme mission operations (Hillman et al., 2008; Teixeira et al., 2012). It is critical to recognize individual preferences, specific environmental challenges, and availability of exercise hardware and exercise options in extreme environments and to examine the volume, intensity, and types of exercises that are most effective toward facilitating psychological health and team performance and cohesion in ICE settings.

Similar to other biologically oriented literature bases, fatigue and sleep are a robust area of research at the individual level, but there is a notable dearth of research at the team level (Chabal et al., 2018; Banks et al., 2019). Empirical studies are needed to predict likely effects of an individual on a team, for example, a fatigued individual exhibiting poor problem solving during a team task would likely delay or result in a non-optimal solution for the team. However, the types of tasks and situations in which teams may be able to mitigate the fatigued state of a member are unknown. In a tightly coupled system, each team member that is not operating at full capacity will have a disproportionate influence on the team outcomes. Many industries make use of validated biomathematical models of fatigue (Van Dongen, 2004) to determine how much sleep and during what time of day sleep is needed to support safety and performance. Relatedly, the IMOI model allows researchers a starting point to systematically examine fatigue as an individual input variable affecting all parts of the model. Integration of these models, along with the integration of additional biological variables, would offer organizations more robust scheduling of teams and timely countermeasure intervention for sustained performance. Furthermore, health management systems, employed across many organizations in many industries to manage the safety and well-being of employees and customers, are currently directed at the individual or organizational policy level and do not include an integrated, comprehensive approach incorporating all behavioral biology topics discussed in this article. These systems would also benefit from leveraging team factors (e.g., backup and supporting behaviors that provide team members the skills to recognize decrements in oneself and others) and take actions to implement countermeasures that support the team member as well as the safety, performance, and functioning of the whole team. Quantification of the success of these programs incorporating team factors and using multi-level experimental designs allows understanding for how teams may best be leveraged to prevent and mitigate negative effects stemming from the multitude of biological causes.

Finally, researchers and practitioners alike in the field of habitability and human factor design may benefit from research that provides a better understanding of the risk of the compounding needs of biological factors in affecting team-related processes and outcomes to provide improved countermeasures within habitat and equipment design for isolated, confined, and extreme environments. More research is needed as to the acute and chronic neurobiological reactions in the brain and other body systems that may be influenced by the physical environment. The physical environment may also directly influence team processes and team and individual outcomes by engendering cohesion and limiting conflict with adequate space and design in which to perform team tasks and recreation, as well as provide individual refuge and privacy. More generally, the duration of living and working in such an environment will exacerbate the effects of environmental stressors; however, the nature of that dynamic relationship over extreme long durations such as a Mars mission is not known. Determining psychological thresholds for tolerance of habitat and systems design variations for missions of varying durations will enable engineers and mission planners to meet the needs for different mission profiles.

## Conclusions and the Path Forward

Over the course of this selective review, it is clear that multidisciplinary science for understanding teams in ICE environments is both a valuable endeavor to move the field forward and a daunting challenge. However, there are many existing structural and scientific integration efforts that may facilitate future research and applications. The first key is forming interdisciplinary research partnerships. These may be accomplished through top-down approaches as policymakers and research funding entities release calls for appropriately funded multidisciplinary research. These organizations may also proactively offer support and guidance to multidisciplinary research teams related to methods of communicating and collaborating between teams with different field-specific norms and languages. For example, the National Institutes of Health’s (NIH) National Cancer Institute hosts a Team Science Toolkit that enables multidisciplinary teams to overcome common hurdles in partnering with others from disparate fields^2^. Creating research questions that are fundamentally multidisciplinary and soliciting proposals with experts from several areas of expertise will prompt researchers in these fields to reach out beyond their typical circles to form new partnerships. Most of this funding originates from government agencies such as the NIH or the National Science Foundation (NSF), which also provide funding opportunities for social neuroscience through their Social, Behavioral, and Economic Sciences (SBE), Behavioral and Cognitive Sciences (BCS), and Social and Economic Sciences (SES) programs. Defense agencies and other organizations that rely on ICE operations (e.g., transoceanic shipping, energy sector, polar research agencies) also have an interest in optimizing team performance and functioning over long durations. Military operations with units such as those deployed in the field and on ships and submarines more akin to the closed systems of spaceflight would likely benefit from integrated approaches to team science and countermeasure development (Goodwin et al., 2018). Optimization of soldier (i.e., the individual-level system) and unit (i.e., the team-level system) performance while on deployment (i.e., the team-in-the-environment system) drives leaders to consider the whole soldier, creating an environment that is conducive to exploring multidisciplinary, cutting-edge research. Researchers should seek out these organizations’ calls for proposals.

From a bottom-up approach, researchers can design experiments that address multiple fields. For example, biomarkers collected as part of an exercise protocol to understand recovery times for different exercise prescriptions may be analyzed for stress hormones that are of interest to psychological researchers. Team researchers may also be able to observe subsequent team interactions following these exercise episodes to understand other interpersonal outcomes of different exercise routines, informing exercise countermeasures that may benefit the physical and psychological health of team members. This research study may be further broadened as sleep and fatigue researchers collect data related to pre- and post-exercise fatigue and sleep needs related to different exercise protocols and nutritional inputs, given varying levels of stress hormones, and so on. The complexity of this type of research also demands careful thinking about research design, sample size and statistical power, and leveraging already existing multidisciplinary datasets for initial exploratory analyses and hypothesis generation such as the NASA Life Sciences Data Archive^3^. Using existing data is one way to minimize costs. For large-scale experiments, such as what is conducted in spaceflight mission simulation analogs with dozens of investigators examining many different factors for the same set of participants, data-sharing agreements between investigator teams from different fields may allow planned multidisciplinary collaboration or hold potential for integrated *post hoc* analyses. As time and resources for research are not unlimited, collaborative integration also offers a cost-effective approach to conducting research.

Team research is especially challenging in operational environments due to the sample size problem; that is, each team may be composed of several individuals, but that team is just an *n* of 1 for any team-level variable. Layering research questions from several fields may require large sample sizes, which is multiplied by the need for sufficiently powered team-level data. Integrated data-mining and application of advanced analytical techniques capable of processing “big data” (e.g., machine learning) may provide findings related to the understudied intersection of different fields and other risks to team functioning (Lazer et al., 2009; Goswami et al., 2013; Luciano et al., 2018)^4^. Also, agent-based modeling experts can parameterize complex, integrated, multidisciplinary models with large-scale existing data. Using agent-based models to conduct virtual experiments allows for investigation of many different specific scenarios, which would otherwise require large numbers of research participants (Epstein, 2006). Current supercomputers, many available from government organizations to any researcher with necessary research approvals and funding, allow this type of data analysis to occur in a matter of hours or days for tens of thousands of virtually simulated experiments. Integrating data across multiple measurement methods and tools supports the identification of the most efficient, yet valid, method of measuring each variable of interest, reducing overall measurement burden on study participants, which is a concern for teams in operational environments.

A multidisciplinary approach to sustaining healthy individual and team performance, well-being, and social interactions may realize more efficiencies and effectiveness when monitoring the team and implementing countermeasures. Integrated monitoring and analysis may help the team and support personnel obtain comprehensive and more accurate assessments of team performance and functioning, individual health and well-being, and identify changing effects on the individuals within the team over time. Multi-pronged interventions may be more effective. For example, if the team collectively is fatigued due to an unexpected emergency waking them in the middle of the night, a multidisciplinary countermeasure package may address how the team may be rescheduled to allow recovery sleep, the design of the sleep environment for adequate privacy and lighting to support sleep, and what foods will enable sleep and provide more sustained energy upon waking so that they are able to recover and perform, etc., without any one countermeasure imposing an unacceptable or disruptive burden. Additionally, understanding each individual team member’s unique systems and needs within a proactively individualized medicine approach (Evans and Relling, 2004; Topol, 2014) may allow countermeasures to be tailored and implemented at both the individual and team levels. Ultimately, the complexity in addressing the multiple pathways that increase risks to individual and team behavioral health and performance is challenging for researchers and practitioners alike. However, multiple pathways that increase risk also provide multiple pathways to reduce risk for teams who work, live, serve, and explore in extreme environments.

## Author Contributions

PR and LL conceived the project and designed the review. PR, LL, GD, MD, AW, MG, and SZ wrote the paper. All authors made substantial contributions and reviewed and approved the completed manuscript.

### Conflict of Interest

The authors declare that the research was conducted in the absence of any commercial or financial relationships that could be construed as a potential conflict of interest.

## Footnotes

^1^https://www.nimh.nih.gov/research-priorities/rdoc/index.shtml

^2^https://www.teamsciencetoolkit.cancer.gov/Public/ToolkitTeam.aspx

^3^https://lsda.jsc.nasa.gov/

^4^https://www.darpa.mil/work-with-us/ai-next-campaign

## References

[ref1] Agureev A. N. Afonin B. V. Sedova E. A. Solovieva A. A. Valuev V. A. Sidorenko L. A. (2017). Nutritional status in the experiment with 105-day isolation as the first phase of the Mars-500 project. Hum. Physiol. 43, 793–801. 10.1134/S0362119717070027 26934786

[ref2] Altman I. (1977). Privacy regulation: culturally universal or culturally specific? J. Soc. Issues 33, 66–84. 10.1111/j.1540-4560.1977.tb01883.x

[ref3] Altun A. Ugur-Altun B. (2007). Melatonin: therapeutic and clinical utilization. Int. J. Clin. Pract. 61, 835–845. 10.1111/j.1742-1241.2006.01191.x 17298593

[ref4] Amico J. A. Tenicela R. Johnston J. Robinson A. G. (1983). A time-dependent peak of oxytocin exists in cerebrospinal fluid but not in plasma of humans. J. Clin. Endocrinol. Metab. 57, 947–951. 10.1210/jcem-57-5-947, PMID: 6619269

[ref5] Andrews P. W. Bharwani A. Lee K. R. Fox M. Thomson J. A. Jr. (2015). Is serotonin an upper or a downer? The evolution of the serotonergic system and its role in depression and the antidepressant response. Neurosci. Biobehav. Rev. 51, 164–188. 10.1016/j.neubiorev.2015.01.018, PMID: 25625874

[ref6] Anker P. (2005). The ecological colonization of space. Environ. Hist. 10, 239–268. 10.1093/envhis/10.2.239

[ref7] Ashkanasy N. M. Becker W. J. Waldman D. A. (2014). Neuroscience and organizational behavior: avoiding both neuro-euphoria and neuro-phobia. J. Organ. Behav. 35, 909–919. 10.1002/job.1952

[ref8] Banks S. Landon L. B. Dorrian J. Waggoner L. B. Centofanti S. A. Roma P. G. (2019). Effects of fatigue on teams and their role in 24/7 operations. Sleep Med. Rev. 48:101216. 10.1016/j.smrv.2019.101216 31630015

[ref9] Baranski J. V. Thompson M. M. Lichacz F. M. McCann C. Gil V. Pastò L. (2007). Effects of sleep loss on team decision making: motivational loss or motivational gain? Hum. Factors 49, 646–660. 10.1518/001872007x215728 17702216

[ref10] Barger L. K. Flynn-Evans E. E. Kubey A. Walsh L. Ronda J. M. Wang W.. (2014). Prevalence of sleep deficiency and use of hypnotic drugs in astronauts before, during, and after spaceflight: an observational study. Lancet Neurol. 13, 904–912. 10.1016/S1474-4422(14)70122-X, PMID: 25127232 PMC4188436

[ref11] Barrett E. Ross R. P. O'Toole P. W. Fitzgerald G. F. Stanton C. (2012). γ-Aminobutyric acid production by culturable bacteria from the human intestine. J. Appl. Microbiol. 113, 411–417. 10.1111/j.1365-2672.2012.05344.x, PMID: 22612585

[ref12] Bartz J. A. Zaki J. Bolger N. Ochsner K. N. (2011). Social effects of oxytocin in humans: context and person matter. Trends Cogn. Sci. 15, 301–309. 10.1016/j.tics.2011.05.002, PMID: 21696997

[ref13] Basner M. Dinges D. F. Mollicone D. Ecker A. Jones C. W. Hyder E. C.. (2013). Mars 520-d mission simulation reveals protracted crew hypokinesis and alterations of sleep duration and timing. Proc. Natl. Acad. Sci. USA 110, 2635–2640. 10.1073/pnas.1212646110, PMID: 23297197 PMC3574912

[ref14] Becker W. J. Cropanzano R. (2010). Organizational neuroscience: the promise and prospects of an emerging discipline. J. Organ. Behav. 31, 1055–1059. 10.1002/job.668

[ref540] Bender H. E. Fracchia J. (1971). Designer’s handbook: Environmental planning for group stability. NASA Technical Report for Contract NAS 9-10998. Houston, TX: NASA Johnson Space Center.

[ref15] Berntson G. G. Bigger J. T. Eckberg D. L. Grossman P. Kaufmann P. G. Malik M.. (1997). Heart rate variability: origins, methods, and interpretive caveats. Psychophysiology 34, 623–648. 10.1111/j.1469-8986.1997.tb02140.x, PMID: 9401419

[ref16] Berridge K. C. Kringelbach M. L. (2015). Pleasure systems in the brain. Neuron 86, 646–664. 10.1016/j.neuron.2015.02.018, PMID: 25950633 PMC4425246

[ref17] Berryman J. W. (2010). Exercise is medicine: a historical perspective. Curr. Sports Med. Rep. 9, 195–201. 10.1249/JSR.0b013e3181e7d86d, PMID: 20622536

[ref18] Bienenstock J. Kunze W. A. Forsythe P. (2018). Disruptive physiology: olfaction and the microbiome–gut–brain axis. Biol. Rev. 93, 390–403. 10.1111/brv.12348, PMID: 28675687

[ref19] Boccia M. L. Petrusz P. Suzuki K. Marson L. Pedersen C. A. (2013). Immunohistochemical localization of oxytocin receptors in human brain. Neuroscience 253, 155–164. 10.1016/j.neuroscience.2013.08.048 24012742

[ref20] Brady J. V. (1990). Toward applied behavior analysis of life aloft. Behav. Sci. 35, 11–23. 10.1002/bs.3830350103, PMID: 11538209

[ref21] Brady J. V. (2005). Behavioral health: the propaedeutic requirement. Aviat. Space Environ. Med. 76, B13–B24. Available at: https://www.ingentaconnect.com/content/asma/asem/2005/00000076/a00106s1/art00003 15943190

[ref22] Byers A. L. Yaffe K. (2011). Depression and risk of developing dementia. Nat. Rev. Neurol. 7, 323–331. 10.1038/nrneurol.2011.60, PMID: 21537355 PMC3327554

[ref23] Celentano J. Amorelli D. Freeman G. (1963). Establishing a habitability index for space stations and planetary bases. Paper presented at the Manned Space Laboratory Conference.

[ref24] Chabal S. Welles R. Haran F. J. Markwald R. (2018). Effects of sleep and fatigue on teams in a submarine environment. Undersea Hyperb. Med. 45, 257–272. 10.22462/05.06.2018.2 30028913

[ref25] Chang Y. K. Labban J. D. Gapin J. I. Etnier J. L. (2012). The effects of acute exercise on cognitive performance: a meta-analysis. Brain Res. 1453, 87–101. 10.1016/j.brainres.2012.02.068, PMID: 22480735

[ref26] Checinska A. Probst A. J. Vaishampayan P. White J. R. Kumar D. Stepanov V. G. (2015). Microbiomes of the dust particles collected from the international space station and spacecraft assembly facilities. Microbiome 3:50. 10.1186/s40168-015-0116-3 26502721 PMC4624184

[ref27] Clark L. A. Cuthbert B. Lewis-Fernández R. Narrow W. E. Reed G. M. (2017). Three approaches to understanding and classifying mental disorder: ICD-11, DSM-5, and the National Institute of Mental Health’s Research Domain Criteria (RDoC). Psychol. Sci. Public Interest 18, 72–145. 10.1177/1529100617727266, PMID: 29211974

[ref28] Cohen D. A. Wang W. Wyatt J. K. Kronauer R. E. Dijk D. J. Czeisler C. A.. (2010). Uncovering residual effects of chronic sleep loss on human performance. Sci. Transl. Med. 2:14ra3. 10.1126/scitranslmed.3000458, PMID: 20371466 PMC2892834

[ref29] Cohrs S. (2008). Sleep disturbances in patients with schizophrenia. CNS Drugs 22, 939–962. 10.2165/00023210-200822110-00004, PMID: 18840034

[ref30] Cooney G. Dwan K. Mead G. (2014). Exercise for depression. J. Am. Med. Assoc. 311, 2432–2433. 10.1001/jama.2014.4930 24938566

[ref31] Cotman C. W. Berchtold N. C. Christie L. A. (2007). Exercise builds brain health: key roles of growth factor cascades and inflammation. Trends Neurosci. 30, 464–472. 10.1016/j.tins.2007.06.011, PMID: 17765329

[ref32] Cuthbert B. N. Kozak M. J. (2013). Constructing constructs for psychopathology: the NIMH research domain criteria. J. Abnorm. Psychol. 122, 928–937. 10.1037/a0034028, PMID: 24016027

[ref33] David L. A. Maurice C. F. Carmody R. N. Gootenberg D. B. Button J. E. Wolfe B. E.. (2014). Diet rapidly and reproducibly alters the human gut microbiome. Nature 505, 559–563. 10.1038/nature12820, PMID: 24336217 PMC3957428

[ref34] De Dreu C. K. Greer L. L. Van Kleef G. A. Shalvi S. Handgraaf M. J. (2011). Oxytocin promotes human ethnocentrism. Proc. Natl. Acad. Sci. USA 108, 1262–1266. 10.1073/pnas.1015316108, PMID: 21220339 PMC3029708

[ref35] DeChurch L. Wang W. Harris A. Contractor N. (2018). Mapping the modern science of teams. Presented at the Interdisciplinary Network for Group Research (INGRoup) Annual Conference, Bethesda, MD.

[ref36] DeCroon E. Sluiter J. Kuijer P. P. Frings-Dresen M. (2005). The effect of office concepts on worker health and performance: a systematic review of the literature. Ergonomics 48, 119–134. 10.1080/00140130512331319409 15764312

[ref37] Delignières D. Marcellini A. Brisswalter J. Legros P. (1994). Self-perception of fitness and personality traits. Percept. Mot. Skills 78, 843–851. 10.2466/pms.1994.78.3.843 8084701

[ref38] Derntl B. Hack R. L. Kryspin-Exner I. Habel U. (2013). Association of menstrual cycle phase with the core components of empathy. Horm. Behav. 63, 97–104. 10.1016/j.yhbeh.2012.10.009, PMID: 23098806 PMC3549494

[ref39] Desbonnet L. Garrett L. Clarke G. Bienenstock J. Dinan T. G. (2008). The probiotic Bifidobacteria infantis: an assessment of potential antidepressant properties in the rat. J. Psychiatr. Res. 43, 164–174. 10.1016/j.jpsychires.2008.03.009, PMID: 18456279

[ref40] Deuster P. A. Silverman M. N. (2013). Physical fitness: a pathway to health and resilience. U.S. Army Med. Dep. J. 24–35. PMID: 24146240

[ref41] Dinan T. G. Cryan J. F. (2017). Microbes, immunity, and behavior: psychoneuroimmunology meets the microbiome. Neuropsychopharmacology 42, 178–192. 10.1038/npp.2016.103, PMID: 27319972 PMC5143479

[ref42] Dinan T. G. Stanton C. Cryan J. F. (2013). Psychobiotics: a novel class of psychotropic. Biol. Psychiatry 74, 720–726. 10.1016/j.biopsych.2013.05.001, PMID: 23759244

[ref43] Douglas G. L. Cooper M. Bermudez-Aguirre D. Sirmons T. (2016). Risk of performance decrement and crew illness due to an inadequate food system. Houston, TX: NASA.

[ref44] Douglas G. L. Voorhies A. A. (2017). Evidence based selection of probiotic strains to promote astronaut health or alleviate symptoms of illness on long duration spaceflight missions. Benefic. Microbes 8, 727–737. 10.3920/BM2017.0027, PMID: 28760005

[ref45] Dowlati Y. Herrmann N. Swardfager W. Liu H. Sham L. Reim E. K.. (2010). A meta-analysis of cytokines in major depression. Biol. Psychiatry 67, 446–457. 10.1016/j.biopsych.2009.09.033, PMID: 20015486

[ref46] Dubocovich M. L. (2007). Melatonin receptors: role on sleep and circadian rhythm regulation. Sleep Med. 8, 34–42. 10.1016/j.sleep.2007.10.007, PMID: 18032103

[ref47] Ebling F. J. (1996). The role of glutamate in the photic regulation of the suprachiasmatic nucleus. Prog. Neurobiol. 50, 109–132. 10.1016/S0301-0082(96)00032-9, PMID: 8971980

[ref48] Eckstein M. Scheele D. Weber K. Stoffel-Wagner B. Maier W. Hurlemann R. (2014). Oxytocin facilitates the sensation of social stress. Hum. Brain Mapp. 35, 4741–4750. 10.1002/hbm.22508, PMID: 24659430 PMC6869318

[ref49] Edwards D. A. Wetzel K. Wyner D. R. (2006). Intercollegiate soccer: saliva cortisol and testosterone are elevated during competition, and testosterone is related to status and social connectedness with teammates. Physiol. Behav. 87, 135–143. 10.1016/j.physbeh.2005.09.007, PMID: 16233905

[ref50] Eisenegger C. Haushofer J. Fehr E. (2011). The role of testosterone in social interaction. Trends Cogn. Sci. 15, 263–271. 10.1016/j.tics.2011.04.008, PMID: 21616702

[ref51] Eisenegger C. Naef M. Snozzi R. Heinrichs M. Fehr E. (2010). Prejudice and truth about the effect of testosterone on human bargaining behaviour. Nature 463, 356–359. 10.1038/nature08711, PMID: 19997098

[ref52] Emurian H. H. Canfield K. Roma P. G. Gasior E. D. Brinson Z. S. Hienz R. D. (2009). Behavioral systems management of confined microsocieties: an agenda for research and applications. Proceedings of the 39th International Conference on Environmental Systems (paper number 2009-01-2423), Warrendale, PA, SAE International.

[ref53] Epstein J. M. (2006). Generative social science: Studies in agent-based computational modeling. Princeton, NJ: Princeton University Press.

[ref54] Evans W. E. Relling M. V. (2004). Moving towards individualized medicine with pharmacogenomics. Nature 429, 464–468. 10.1038/nature02626, PMID: 15164072

[ref55] Feldman R. (2012). Oxytocin and social affiliation in humans. Horm. Behav. 61, 380–391. 10.1016/j.yhbeh.2012.01.008, PMID: 22285934

[ref56] Fiore S. M. Wiltshire T. J. Sanz E. J. Pajank M. E. Center J. S. (2015). Critical team cognitive processes for long-duration exploration missions. NASA TM-2015-218583.

[ref57] Fleming I. Baum A. Weiss L. (1987). Social density and perceived control as mediators of crowding stress in high-density residential neighborhoods. J. Pers. Soc. Psychol. 52, 899–906.

[ref58] Fletcher A. Wesensten N. J. Kandelaars K. Balkin T. J. (2012). Measuring and predicting sleep and performance during military operations. Silver Spring, MD: Walter Reed Army Institute of Research.

[ref59] Foxall G. R. (2014a). Cognitive requirements of competing neuro-behavioral decision systems: some implications of temporal horizon for managerial behavior in organizations. Front. Hum. Neurosci. 8:184. 10.3389/fnhum.2014.00184 24744719 PMC3978328

[ref60] Foxall G. R. (2014b). The marketing firm and consumer choice: implications of bilateral contingency for levels of analysis in organizational neuroscience. Front. Hum. Neurosci. 8:472. 10.3389/fnhum.2014.00472 25071506 PMC4078247

[ref61] Frankland P. W. Bontempi B. (2005). The organization of recent and remote memories. Nat. Rev. Neurosci. 6, 119–130. 10.1038/nrn1607, PMID: 15685217

[ref62] Fuster J. M. (2001). The prefrontal cortex—an update: time is of the essence. Neuron 30, 319–333. 10.1016/S0896-6273(01)00285-9, PMID: 11394996

[ref63] Gander P. H. Purnell H. M. Garden A. Woodward A. (2007). Work patterns and fatigue-related risk among junior doctors. Occup. Environ. Med. 64, 733–738. 10.1136/oem.2006.030916, PMID: 17387138 PMC2078416

[ref64] Garrett-Bakelman F. E. Darshi M. Green S. J. Gur R. C. Lin L. Macias B. R.. (2019). The NASA twins study: a multidimensional analysis of a year-long human spaceflight. Science 364:eaau8650. 10.1126/science.aau8650, PMID: 30975860 PMC7580864

[ref65] Gianaros P. J. Kuan D. C. H. Marsland A. L. Sheu L. K. Hackman D. A. Miller K. G.. (2015). Community socioeconomic disadvantage in midlife relates to cortical morphology via neuroendocrine and cardiometabolic pathways. Cereb. Cortex 27, 460–473. 10.1093/cercor/bhv233, PMID: 26498832 PMC5897839

[ref66] Gitelson J. I. Lisovsky G. M. MacElroy R. D. (2003). Manmade closed ecological systems. New York: CRC Press.

[ref67] Golden S. J. Chang C. H. Kozlowski S. W. (2018). Teams in isolated, confined, and extreme (ICE) environments: review and integration. J. Organ. Behav. 39, 701–715. 10.1002/job.2288

[ref68] Goodwin G. F. Blacksmith N. Coats M. R. (2018). The science of teams in the military: contributions from over 60 years of research. Am. Psychol. 73, 322–333. 10.1037/amp0000259, PMID: 29792451

[ref69] Goswami N. Batzel J. J. Clément G. Stein T. P. Sharp M. K. Blaber A. P.. (2013). Maximizing information from space data resources: a case for expanding integration between research disciplines. Eur. J. Appl. Physiol. 113, 1645–1654. 10.1007/s00421-012-2507-5, PMID: 23073848

[ref70] Goswami N. Roma P. G. De Boever P. Clément G. Hargens A. R. Loeppky J. A. (2012). Using the moon as a high-fidelity analogue environment to study biological and behavioural mechanisms of long-duration space exploration. Planet. Space Sci. 74, 111–120. 10.1016/j.pss.2012.07.030

[ref71] Grillner S. (2015). Action: the role of motor cortex challenged. Curr. Biol. 25, R508–R511. 10.1016/j.cub.2015.04.023, PMID: 26079084

[ref72] Groeger J. A. Viola A. U. Lo J. C. von Schantz M. Archer S. N. Dijk D. J. (2008). Early morning executive functioning during sleep deprivation is compromised by a PERIOD3 polymorphism. Sleep 31, 1159–1167. 10.5665/sleep/31.8.1159, PMID: 18714788 PMC2542962

[ref73] Grosso G. Galvano F. Marventano S. Malaguarnera M. Bucolo C. Drago F.. (2014). Omega-3 fatty acids and depression: scientific evidence and biological mechanisms. Oxid. Med. Cell. Longev. 2014:313570. 10.1155/2014/313570, PMID: 24757497 PMC3976923

[ref74] Haus E. (2007). Chronobiology in the endocrine system. Adv. Drug Deliv. Rev. 59, 985–1014. 10.1016/j.addr.2007.01.001, PMID: 17804113

[ref75] Heinrichs M. Baumgartner T. Kirschbaum C. Ehlert U. (2003). Social support and oxytocin interact to suppress cortisol and subjective responses to psychosocial stress. Biol. Psychiatry 54, 1389–1398. 10.1016/S0006-3223(03)00465-7, PMID: 14675803

[ref76] Heller A. S. Johnstone T. Shackman A. J. Light S. N. Peterson M. J. Kolden G. G.. (2009). Reduced capacity to sustain positive emotion in major depression reflects diminished maintenance of fronto-striatal brain activation. Proc. Natl. Acad. Sci. USA 106, 22445–22450. 10.1073/pnas.0910651106, PMID: 20080793 PMC2796908

[ref77] Hillman C. H. Erickson K. I. Kramer A. F. (2008). Be smart, exercise your heart: exercise effects on brain and cognition. Nat. Rev. Neurosci. 9, 58–65. 10.1038/nrn2298, PMID: 18094706

[ref78] Howren M. B. Lamkin D. M. Suls J. (2009). Associations of depression with C-reactive protein, IL-1, and IL-6: a meta-analysis. Psychosom. Med. 71, 171–186. 10.1097/PSY.0b013e3181907c1b, PMID: 19188531

[ref79] Ilgen D. R. Hollenbeck J. R. Johnson M. Jundt D. (2005). Teams in organizations: from input-process-output models to IMOI models. Annu. Rev. Psychol. 56, 517–543. 10.1146/annurev.psych.56.091103.070250, PMID: 15709945

[ref80] Jaggar M. Fanibunda S. E. Ghosh S. Duman R. S. Vaidya V. A. (2019). “The neurotrophic hypothesis of depression revisited: new insights and therapeutic implications” in Neurobiology of depression. eds. Quevedo J. Carvalho A. F. Zarate C. A. (Cambridge, MA: Academic Press), 43–62.

[ref81] JuS. Y.LeeY. J.JeongS. N. (2013). Serum 25-hydroxyvitamin D levels and the risk of depression: a systematic review and meta-analysis. J. Nutr. Health Aging 17, 447–455. 10.1007/s12603-012-0418-023636546

[ref82] Karlin R. A. Rosen L. S. Epstein Y. M. (1979). Three into two doesn’t go: a follow-up on the effects of overcrowded dormitory rooms. Personal. Soc. Psychol. Bull. 5, 391–395.

[ref83] Kearney A. R. (2016). Team health and performance in spaceflight habitats. NASA/TM-2016-219274.

[ref84] Killeen P. R. (2018). The futures of experimental analysis of behavior. Behav. Anal. Res. Pract. 18, 124–133. 10.1037/bar0000100

[ref85] Kim J. S. de La Serre C. B. (2018). Diet, gut microbiota composition and feeding behavior. Physiol. Behav. 192, 177–181. 10.1016/j.physbeh.2018.03.026, PMID: 29605585

[ref86] Kim T. W. Jeong J. H. Hong S. C. (2015). The impact of sleep and circadian disturbance on hormones and metabolism. Int. J. Endocrinol. 2015:591729. 10.1155/2015/591729, PMID: 25861266 PMC4377487

[ref541] Klerman E. B. Dijk D. J. (2005). Interindividual variation in sleep duration and its association with sleep debt in young adults. Sleep 28, 1253–1259. 10.1093/sleep/28.10.1253 16295210 PMC1351048

[ref87] Kozlowski S. W. J. Chang C. H. Perry S. B. Pearce M. Dixon A. J. Santoro J. M. (2015). Capturing team process dynamics. Presented at the Annual Conference for the Society for Industrial/Organizational Psychologists, Philadelphia, PA.

[ref88] Kozlowski S. W. J. Ilgen D. R. (2006). Enhancing the effectiveness of work groups and teams. Psychol. Sci. Public Interest 7, 77–124. 10.1111/j.1529-1006.2006.00030.x 26158912

[ref89] Kozlowski S. W. J. Klein K. J. (2000). “A multilevel approach to theory and research in organizations: contextual, temporal, and emergent processes” in Multilevel theory, research and methods in organizations: Foundations, extensions, and new directions (San Francisco, CA: Jossey-Bass), 3–90.

[ref90] Kraemer W. J. Flanagan S. D. Volek J. S. Nindl B. C. Vingren J. L. Dunn-Lewis C.. (2013). Resistance exercise induces region-specific adaptations in anterior pituitary gland structure and function in rats. J. Appl. Physiol. 115, 1641–1647. 10.1152/japplphysiol.00687.2013, PMID: 24092688

[ref91] Krakauer J. W. Ghazanfar A. A. Gomez-Marin A. MacIver M. A. Poeppel D. (2017). Neuroscience needs behavior: correcting a reductionist bias. Neuron 93, 480–490. 10.1016/j.neuron.2016.12.041, PMID: 28182904

[ref92] Landon L. B. Rokholt C. Slack K. J. Pecena Y. (2017). Selecting astronauts for long-duration exploration missions: considerations for team performance and functioning. Reach 5, 33–56. 10.1016/j.reach.2017.03.002

[ref93] Landon L. B. Slack K. J. Barrett J. D. (2018). Teamwork and collaboration in long-duration space missions: going to extremes. Am. Psychol. 73, 563–575. 10.1037/amp0000260, PMID: 29792468

[ref94] Laurence G. A. Fried Y. Slowik L. H. (2013). “My space”: a moderated mediation model of the effect of architectural and experienced privacy and workspace personalization on emotional exhaustion at work. J. Environ. Psychol. 36, 144–152. 10.1016/j.jenvp.2013.07.011

[ref95] Lazer D. Pentland A. S. Adamic L. Aral S. Barabasi A. L. Brewer D.. (2009). Life in the network: the coming age of computational social science. Science 323, 721–723. 10.1126/science.1167742, PMID: 19197046 PMC2745217

[ref96] Lebow M. A. Chen A. (2016). Overshadowed by the amygdala: the bed nucleus of the stria terminalis emerges as key to psychiatric disorders. Mol. Psychiatry 21, 450–463. 10.1038/mp.2016.1, PMID: 26878891 PMC4804181

[ref97] LeDoux J. (2007). The amygdala. Curr. Biol. 17, R868–R874. 10.1016/j.cub.2007.08.005, PMID: 17956742

[ref98] Lee N. Senior C. Butler M. J. (2012). The domain of organizational cognitive neuroscience: theoretical and empirical challenges. J. Manag. 38, 921–931. 10.1177/0149206312439471

[ref99] Lemon R. N. (2008). Descending pathways in motor control. Annu. Rev. Neurosci. 31, 195–218. 10.1146/annurev.neuro.31.060407.125547, PMID: 18558853

[ref100] Lemos J. R. (2012). “Magnocellular neurons” in Encyclopedia of life sciences. ed. Kendall S. K. (Chichester: John Wiley & Sons Ltd.), 19–21.

[ref101] Lengyel D. M. Newman J. S. (2014). International space station lessons learned for space exploration. NASA Public Lessons Learned System Database, Entry, 12603.

[ref102] Leonard B. E. (2005). The HPA and immune axes in stress: the involvement of the serotonergic system. Eur. Psychiatry 20, S302–S306. 10.1016/s0924-9338(05)80180-4 16459240

[ref103] Li L. Su Q. Xie B. Duan L. Zhao W. Hu D.. (2016). Gut microbes in correlation with mood: case study in a closed experimental human life support system. Neurogastroenterol. Motil. 28, 1233–1240. 10.1111/nmo.12822, PMID: 27027909

[ref104] Luciano M. M. Mathieu J. E. Park S. Tannenbaum S. I. (2018). A fitting approach to construct and measurement alignment: the role of big data in advancing dynamic theories. Organ. Res. Methods 21, 592–631. 10.1177/1094428117728372

[ref105] Lydiard R. B. (2003). The role of GABA in anxiety disorders. J. Clin. Psychiatry 64, 21–27. Available at: https://www.psychiatrist.com/jcp/article/pages/2003/v64s03/v64s0304.aspx 12662130

[ref106] Lynch M. A. (2004). Long-term potentiation and memory. Physiol. Rev. 84, 87–136. 10.1152/physrev.00014.2003 14715912

[ref107] Marriott B. M. (1995). Not eating enough: Overcoming underconsumption of military operational rations. Washington, DC: National Academies Press. 25121269

[ref108] Mathieu J. E. Kukenberger M. R. D’innocenzo L. Reilly G. (2015). Modeling reciprocal team cohesion–performance relationships, as impacted by shared leadership and members’ competence. J. Appl. Psychol. 100, 713–734. 10.1037/a0038898, PMID: 25751749

[ref109] Maynard M. T. Kennedy D. M. Resick C. J. (2018). Teamwork in extreme environments: lessons, challenges, and opportunities. J. Organ. Behav. 39, 695–700. 10.1002/job.2302

[ref110] McCann J. C. Ames B. N. (2008). Is there convincing biological or behavioral evidence linking vitamin D deficiency to brain dysfunction? FASEB J. 22, 982–1001. 10.1096/fj.07-9326rev, PMID: 18056830

[ref111] McClelland J. L. McNaughton B. L. O'reilly R. C. (1995). Why there are complementary learning systems in the hippocampus and neocortex: insights from the successes and failures of connectionist models of learning and memory. Psychol. Rev. 102, 419–457. 10.1037/0033-295X.102.3.419, PMID: 7624455

[ref112] McGaugh J. L. (1992). “Neuromodulatory systems and the regulation of memory storage” in Neuropsychology of memory. eds. Squire L. R. Butters N. (New York, NY: Guilford Press), 386–401.

[ref113] McMurray I. Connolly H. Preston-Shoot M. Wigley V. (2008). Constructing resilience: social workers’ understandings and practice. Health Soc. Care Community 16, 299–309. 10.1111/j.1365-2524.2008.00778.x 18363698

[ref114] Miller E. K. Cohen J. D. (2001). An integrative theory of prefrontal cortex function. Annu. Rev. Neurosci. 24, 167–202. 10.1146/annurev.neuro.24.1.167, PMID: 11283309

[ref115] Milon H. Decarli B. Adine A. M. Kihm E. (1996). “Food intake and nutritional status during EXEMSI” in Advances in space biology and medicine. Vol. 5. ed. Bonting S. L. (Amsterdam: Elsevier), 79–91. 8814814

[ref116] Mitsuya H. Omata N. Kiyono Y. Mizuno T. Murata T. Mita K.. (2015). The co-occurrence of zinc deficiency and social isolation has the opposite effects on mood compared with either condition alone due to changes in the central norepinephrine system. Behav. Brain Res. 284, 125–130. 10.1016/j.bbr.2015.02.005, PMID: 25680677

[ref117] Mohanty S. Jørgensen J. Nyström M. (2006). Psychological factors associated with habitat design for planetary mission simulators. Space 2006, 1–12. 10.2514/6.2006-7345

[ref118] Molero-Luis M. Serrano M. O’Callaghan M. M. Sierra C. Pérez-Dueñas B. García-Cazorla A.. (2015). Clinical, etiological and therapeutic aspects of cerebral folate deficiency. Expert. Rev. Neurother. 15, 793–802. 10.1586/14737175.2015.1055322, PMID: 26092490

[ref119] Murray M. M. Antonakis J. (2019). An introductory guide to organizational neuroscience. Organ. Res. Methods 22, 6–16. 10.1177/1094428118802621

[ref120] Myers B. Greenwood-VanMeerveld B. (2009). Role of anxiety in the pathophysiology of irritable bowel syndrome: importance of the amygdala. Front. Neurosci. 3:47. 10.3389/neuro.21.002.2009, PMID: 20582274 PMC3112316

[ref121] NASA (2015). Space flight human system standards. Volume 2: human factors, habitability, and environmental health. Rev. A. Available at: https://www.nasa.gov/sites/default/files/atoms/files/nasa-std-3001-vol-2a.pdf (Accessed November 5, 2019).

[ref123] Nestler E. J. Carlezon W. A. Jr. (2006). The mesolimbic dopamine reward circuit in depression. Biol. Psychiatry 59, 1151–1159. 10.1016/j.biopsych.2005.09.018, PMID: 16566899

[ref124] Nohynek L. J. Alakomi H. L. Kähkönen M. P. Heinonen M. Helander I. M. Oksman-Caldentey K. M.. (2006). Berry phenolics: antimicrobial properties and mechanisms of action against severe human pathogens. Nutr. Cancer 54, 18–32. 10.1207/s15327914nc5401_4, PMID: 16800770

[ref125] Obri A. Khrimian L. Karsenty G. Oury F. (2018). Osteocalcin in the brain: from embryonic development to age-related decline in cognition. Nat. Rev. Endocrinol. 14, 174–182. 10.1038/nrendo.2017.181, PMID: 29376523 PMC5958904

[ref126] Ozbay F. Johnson D. C. Dimoulas E. Morgan C. A. III. Charney D. Southwick S. (2007). Social support and resilience to stress: from neurobiology to clinical practice. Psychiatry (Edgemont) 4, 35–40. PMC2921311 20806028

[ref127] Padgett D. A. Glaser R. (2003). How stress influences the immune response. Trends Immunol. 24, 444–448. 10.1016/S1471-4906(03)00173-X, PMID: 12909458

[ref128] Pariante C. M. Lightman S. L. (2008). The HPA axis in major depression: classical theories and new developments. Trends Neurosci. 31, 464–468. 10.1016/j.tins.2008.06.006, PMID: 18675469

[ref129] Patrick R. P. Ames B. N. (2015). Vitamin D and the omega-3 fatty acids control serotonin synthesis and action, part 2: relevance for ADHD, bipolar disorder, schizophrenia, and impulsive behavior. FASEB J. 29, 2207–2222. 10.1096/fj.14-268342, PMID: 25713056

[ref130] Perrow C. (1984). Normal accidents: Living with high risk systems. New York: Basic Books.

[ref131] Phillips M. L. Young A. W. Scott S. Calder A. J. Andrew C. Giampietro V.. (1998). Neural responses to facial and vocal expressions of fear and disgust. Proc. R. Soc. Lond. Ser. B Biol. Sci. 265, 1809–1817. 10.1098/rspb.1998.0506, PMID: 9802236 PMC1689379

[ref132] Pilz L. K. Carissimi A. Oliveira M. A. Francisco A. P. Fabris R. C. Medeiros M. S.. (2018). Rhythmicity of mood symptoms in individuals at risk for psychiatric disorders. Sci. Rep. 8:11402. 10.1038/s41598-018-29348-z, PMID: 30061722 PMC6065390

[ref133] Ploutz-Snyder L. Ryder J. English K. Haddad F. Baldwin K. (2015). Evidence report: Risk of impaired performance due to reduced muscle mass, strength, and endurance. Houston, TX: NASA Johnson Space Center Available at: https://humanresearchroadmap.nasa.gov/Evidence/reports/Muscle.pdf (Accessed November 5, 2019).

[ref134] Reid K. J. Baron K. G. Lu B. Naylor E. Wolfe L. Zee P. C. (2010). Aerobic exercise improves self-reported sleep and quality of life in older adults with insomnia. Sleep Med. 11, 934–940. 10.1016/j.sleep.2010.04.014, PMID: 20813580 PMC2992829

[ref135] Ribeiro S. C. Domingos-Lopes M. F. Stanton C. Ross R. P. Silva C. C. (2018). Production of γ-aminobutyric acid (GABA) by *Lactobacillus otakiensis* and other *Lactobacillus* sp. isolated from traditional Pico cheese. Int. J. Dairy Technol. 71, 1012–1017. 10.1111/1471-0307.12527

[ref136] Roig M. Skriver K. Lundbye-Jensen J. Kiens B. Nielsen J. B. (2012). A single bout of exercise improves motor memory. PLoS One 7:e44594. 10.1371/journal.pone.0044594, PMID: 22973462 PMC3433433

[ref137] Roma P. G. Bedwell W. L. (2017). “Key factors and threats to team dynamics in long-duration extreme environments” in Research on managing groups and teams (vol. 18), team dynamics over time: Advances in theory, methods, and practice. eds. Salas E. Vessey W. B. Landon L. B. (Bingley, UK: Emerald Publishing Limited), 155–187.

[ref138] Roohani N. Hurrell R. Kelishadi R. Schulin R. (2013). Zinc and its importance for human health: an integrative review. J. Res. Med. Sci. 18, 144–157. PMID: 23914218 PMC3724376

[ref139] Rosenwasser A. M. (2009). Functional neuroanatomy of sleep and circadian rhythms. Brain Res. Rev. 61, 281–306. 10.1016/j.brainresrev.2009.08.001, PMID: 19695288

[ref542] Ross H. E. Young L. J. (2009). Oxytocin and the neural mechanisms regulating social cognition and affiliative behavior. Front. Neuroendocrinol. 30, 534–547. 10.1016/j.yfrne.2009.05.004 19481567 PMC2748133

[ref140] Rothhammer V. Borucki D. M. Tjon E. C. Takenaka M. C. Chao C. C. Ardura-Fabregat A.. (2018). Microglial control of astrocytes in response to microbial metabolites. Nature 557, 724–728. 10.1038/s41586-018-0119-x, PMID: 29769726 PMC6422159

[ref141] Salamone J. D. Correa M. Mingote S. M. Weber S. M. (2005). Beyond the reward hypothesis: alternative functions of nucleus accumbens dopamine. Curr. Opin. Pharmacol. 5, 34–41. 10.1016/j.coph.2004.09.004, PMID: 15661623

[ref142] Salas E. Reyes D. L. McDaniel S. H. (2018). The science of teamwork: progress, reflections, and the road ahead. Am. Psychol. 73, 593–600. 10.1037/amp0000334, PMID: 29792470

[ref143] Salgado S. Kaplitt M. G. (2015). The nucleus accumbens: a comprehensive review. Stereotact. Funct. Neurosurg. 93, 75–93. 10.1159/000368279, PMID: 25720819

[ref144] Sampson T. R. Mazmanian S. K. (2015). Control of brain development, function, and behavior by the microbiome. Cell Host Microbe 17, 565–576. 10.1016/j.chom.2015.04.011, PMID: 25974299 PMC4442490

[ref543] Sarter M. Givens B. Bruno J. P. (2001). The cognitive neuroscience of sustained attention: Where top-down meets bottom-up. Brain Res. Rev. 35, 146–160. 10.1016/s0165-0173(01)00044-3, PMID: 11336780

[ref145] Seidel E. M. Silani G. Metzler H. Thaler H. Lamm C. Gur R. C.. (2013). The impact of social exclusion vs. inclusion on subjective and hormonal reactions in females and males. Psychoneuroendocrinology 38, 2925–2932. 10.1016/j.psyneuen.2013.07.021, PMID: 23972943 PMC3863951

[ref146] Shaffer J. A. Edmondson D. Wasson L. T. Falzon L. Homma K. Ezeokoli N.. (2014). Vitamin D supplementation for depressive symptoms: a systematic review and meta-analysis of randomized controlled trials. Psychosom. Med. 76, 190–196. 10.1097/PSY.0000000000000044, PMID: 24632894 PMC4008710

[ref147] Shalvi S. De Dreu C. K. (2014). Oxytocin promotes group-serving dishonesty. Proc. Natl. Acad. Sci. USA 111, 5503–5507. 10.1073/pnas.1400724111, PMID: 24706799 PMC3992689

[ref148] Shan C. Ghosh A. Guo X. Z. Wang S. M. Hou Y. F. Li S. T. (2019). Roles for osteocalcin in brain signalling: implications in cognition-and motor-related disorders. Mol. Brain 12:23. 10.1186/s13041-019-0444-5 30909971 PMC6434857

[ref149] Sian J. Youdim M. B. H. Riederer P. Gerlach M. (1999). “Biochemical anatomy of the basal ganglia and associated neural systems” in Basic neurochemistry: Molecular, cellular and medical aspects. 6th Edn. eds. Siegel G. J. Agranoff B. W. Albers R. W. Fisher S. K. Uhler M. D. (Philadelphia: Lippincott-Raven).

[ref150] Silverman M. N. Deuster P. A. (2014). Biological mechanisms underlying the role of physical fitness in health and resilience. Interface Focus 4:20140040. 10.1098/rsfs.2014.0040, PMID: 25285199 PMC4142018

[ref151] Silverstone S. E. (1997). Food production and nutrition for the crew during the first 2-year closure of biosphere 2. Life Support Biosph. Sci. 4, 167–178. 11542292

[ref152] Smith S. M. Wastney M. E. Morukov B. V. Larina I. M. Nyquist L. E. Abrams S. A. (1999). Calcium metabolism before, during, and after a 3-mo spaceflight: kinetic and biochemical changes. Am. J. Physiol. Regul. Integr. Comp. Physiol. 277, R1–R10. 10.1152/ajpregu.1999.277.1.r1 10409251

[ref153] Šolcová I. P. Šolcová I. Stuchlíková I. Mazehóová Y. (2016). The story of 520 days on a simulated flight to Mars. Acta Astronaut. 126, 178–189. 10.1016/j.actaastro.2016.04.026

[ref154] Squire L. R. (1992). Memory and the hippocampus: a synthesis from findings with rats, monkeys, and humans. Psychol. Rev. 99, 195–231. 10.1037/0033-295X.99.2.195, PMID: 1594723

[ref155] Statton M. A. Encarnacion M. Celnik P. Bastian A. J. (2015). A single bout of moderate aerobic exercise improves motor skill acquisition. PLoS One 10:e0141393. 10.1371/journal.pone.0141393, PMID: 26506413 PMC4624775

[ref156] Stilling R. M. Dinan T. G. Cryan J. F. (2014). Microbial genes, brain & behaviour–epigenetic regulation of the gut–brain axis. Genes Brain Behav. 13, 69–86. 10.1111/gbb.12109, PMID: 24286462

[ref158] Stuster J. (1996). Bold endeavors: Lessons from polar and space exploration. Annapolis, MD: Naval Institute Press. 11543280

[ref157] Stuster J. (2010). Behavioral issues associated with isolation and confinement: review and analysis of astronaut journals. National Aeronautics and Space Administration. NASA/TM-2010-216130.

[ref159] Stuster J. (2016). Behavioral issues associated with long duration space expeditions: review and analysis of astronaut journals. Phase 2 final report. NASA/TM-2016-218603.

[ref160] Supekar K. Kochalka J. Schaer M. Wakeman H. Qin S. Padmanabhan A.. (2018). Deficits in mesolimbic reward pathway underlie social interaction impairments in children with autism. Brain 141, 2795–2805. 10.1093/brain/awy191, PMID: 30016410 PMC6113649

[ref161] Sylvia K. E. Demas G. E. (2018). A gut feeling: microbiome-brain-immune interactions modulate social and affective behaviors. Horm. Behav. 99, 41–49. 10.1016/j.yhbeh.2018.02.001, PMID: 29427583 PMC5880698

[ref162] Teixeira P. J. Carraça E. V. Markland D. Silva M. N. Ryan R. M. (2012). Exercise, physical activity, and self-determination theory: a systematic review. Int. J. Behav. Nutr. Phys. Act. 9, 78. 10.1186/1479-5868-9-78, PMID: 22726453 PMC3441783

[ref163] Tengeler A. C. Kozicz T. Kiliaan A. J. (2018). Relationship between diet, the gut microbiota, and brain function. Nutr. Rev. 76, 603–617. 10.1093/nutrit/nuy016, PMID: 29718511

[ref164] Topol E. J. (2014). Individualized medicine from prewomb to tomb. Cell 157, 241–253. 10.1016/j.cell.2014.02.012, PMID: 24679539 PMC3995127

[ref165] Vadnie C. A. McClung C. A. (2017). Circadian rhythm disturbances in mood disorders: insights into the role of the suprachiasmatic nucleus. Neural Plast. 2017:1504507. 10.1155/2017/1504507, PMID: 29230328 PMC5694588

[ref166] van der Helm E. Gujar N. Walker M. P. (2010). Sleep deprivation impairs the accurate recognition of human emotions. Sleep 33, 335–342. 10.1093/sleep/33.3.335, PMID: 20337191 PMC2831427

[ref167] Van Dongen H. P. A. (2004). Comparison of mathematical model predictions to experimental data of fatigue and performance. Aviat. Space Environ. Med. 75(Suppl. 3), A15–A36. PMID: Available at: https://www.ingentaconnect.com/content/asma/asem/2004/00000075/a00103s1/art00003 15018263

[ref168] Vandewalle G. Archer S. N. Wuillaume C. Balteau E. Degueldre C. Luxen A.. (2009). Functional magnetic resonance imaging-assessed brain responses during an executive task depend on interaction of sleep homeostasis, circadian phase, and PER3 genotype. J. Neurosci. 29, 7948–7956. 10.1523/JNEUROSCI.0229-09.2009, PMID: 19553435 PMC6666044

[ref169] Vuong H. E. Yano J. M. Fung T. C. Hsiao E. Y. (2017). The microbiome and host behavior. Annu. Rev. Neurosci. 40, 21–49. 10.1146/annurev-neuro-072116-031347, PMID: 28301775 PMC6661159

[ref170] Wall R. Cryan J. F. Ross R. P. Fitzgerald G. F. Dinan T. G. Stanton C. (2014). “Bacterial neuroactive compounds produced by psychobiotics” in Microbial endocrinology: The microbiota-gut-brain axis in health and disease. eds. Lyte M. Cryan J. F. (New York, NY: Springer), 221–239. 10.1007/978-1-4939-0897-4_10 24997036

[ref171] Whitmore J. Chaiken S. Fischer J. Harrison R. Harville D. (2008). Sleep loss and complex team performance. Air Force Research Lab Human Effectiveness Directorate Biosciences and Protection Division (No. AFRL-RH-BR-TR-2008-0005).

[ref172] Whitmore M. McGuire K. Margerum S. Thompson S. Allen C. Bowen C. (2013). Evidence report: Risk of incompatible vehicle/habitat design. Houston, TX: NASA Johnson Space Center.

[ref173] Wikoff W. R. Anfora A. T. Liu J. Schultz P. G. Lesley S. A. Peters E. C.. (2009). Metabolomics analysis reveals large effects of gut microflora on mammalian blood metabolites. Proc. Natl. Acad. Sci. USA 106, 3698–3703. 10.1073/pnas.0812874106, PMID: 19234110 PMC2656143

[ref174] Xie L. Kang H. Xu Q. Chen M. J. Liao Y. Thiyagarajan M.. (2013). Sleep drives metabolite clearance from the adult brain. Science 342, 373–377. 10.1126/science.1241224, PMID: 24136970 PMC3880190

[ref175] Xie Y. Yang W. Tang F. Chen X. Ren L. (2015). Antibacterial activities of flavonoids: structure-activity relationship and mechanism. Curr. Med. Chem. 22, 132–149. 10.2174/0929867321666140916113443, PMID: 25245513

[ref176] Yang Y. Raine A. (2009). Prefrontal structural and functional brain imaging findings in antisocial, violent, and psychopathic individuals: a meta-analysis. Psychiatry Res. Neuroimaging 174, 81–88. 10.1016/j.pscychresns.2009.03.012, PMID: 19833485 PMC2784035

[ref177] Yehuda R. (2001). Biology of posttraumatic stress disorder. J. Clin. Psychiatry 62(Suppl. 17), 41–46. Available at: https://www.psychiatrist.com/jcp/article/pages/2001/v62s17/v62s1708.aspx 11495096

[ref178] Zyphur M. J. Narayanan J. Koh G. Koh D. (2009). Testosterone-status mismatch lowers collective efficacy in groups: evidence from a slope-as-predictor multilevel structural equation model. Organ. Behav. Hum. Decis. Process. 110, 70–79. 10.1016/j.obhdp.2009.05.004

